# A nonavalent BODIPY with a multivalent arrangement of α-mannosides enables lectins recognition in fluorescence-based assays

**DOI:** 10.1039/d5cb00190k

**Published:** 2025-12-05

**Authors:** Giacomo Biagiotti, Edvin Purić, Jacopo Tricomi, Janez Mravljak, Stefano Cicchi, Marco Laurati, Yvette van Kooyk, Fabrizio Chiodo, Iztok Urbančič, Marko Anderluh, Barbara Richichi

**Affiliations:** a Department of Chemistry ‘Ugo Schiff’, University of Firenze Via della Lastruccia 3-13 50019 Sesto Fiorentino Italy barbara.richichi@unifi.it; b Department of Pharmaceutical Chemistry, Faculty of Pharmacy, University of Ljubljana Aškerčeva cesta 7 1000 Ljubljana Slovenia Marko.Anderluh@ffa.uni-lj.si; c Department of Molecular Cell Biology and Immunology, Amsterdam UMC, Vrije Universiteit Amsterdam Amsterdam 1081 HV The Netherlands; d Institute of Biomolecular Chemistry, National Research Council (CNR) via Campi Flegrei, 34 80078 Pozzuoli Naples Italy; e Laboratory of Biophysics, Condensed Matter Physics Department, Jožef Stefan Institute Jamova Cesta 39 Ljubljana Slovenia; f Consorzio per lo Sviluppo dei Sistemi a Grande Interfase 50019 Sesto Fiorentino (FI) Firenze Italy

## Abstract

We report here the use of Tris-BODIPY-OH as a scaffold for the multivalent display of sugar heads. A chloroacetyl thioether ligation reaction easily yields mannosylated BODIPYs, named Man_9_-BODIPY and (Man-TEG)_9_-BODIPY, which display nine mannose residues. Regardless of the linker length, both glycoBODIPYs provide an arrangement of mannose heads that allows for proper recognition by the carbohydrate binding domain of concanavalin A (ConA). Moreover, the interactions of Man_9_-BODIPY with relevant human lectins, *i.e.* dendritic cell-specific intercellular adhesion molecule-3-grabbing non-integrin (DC-SIGN) and langerin, were further investigated. The approach proposed is versatile and paves the way for the development of multivalent and fluorescent glyco-BODIPY probes useful to interrogate carbohydrate–lectin interactions in different biological contexts.

## Abstract 2

We report here a multivalent fluorescent glycoprobe useful to interrogate carbohydrate–lectin interactions.

## Graphical abstract

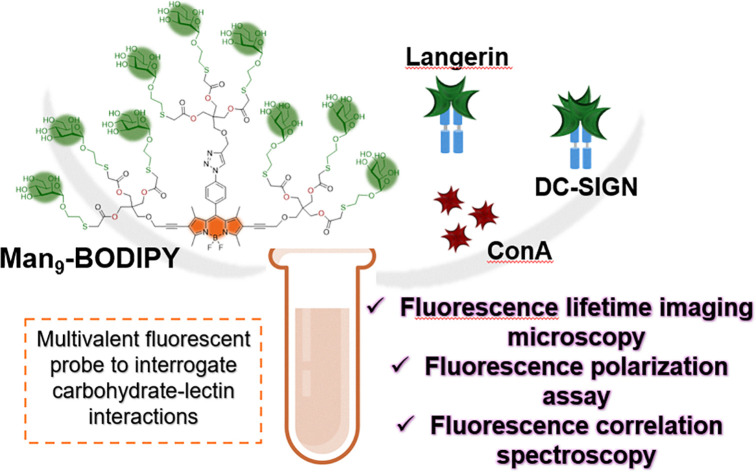

## Introduction

Glyco-BODIPYs have emerged as a promising class of fluorescent and functional compounds that result from the integration of carbohydrates with BODIPY probes.^1,2^ These conjugates exploit the synergic and favorable properties of both components, which are assembled in post-functionalization reactions on pre-formed BODIPY cores using various chemistry (*i.e.* glycosidation reaction, click chemistry and amide/ester formation) or by the introduction of carbohydrate motifs during BODIPY core synthesis.

The BODIPY core provides robust and tunable optical properties, while the carbohydrate moieties serve to improve water solubility, increase biocompatibility, reduce cytotoxicity, and further act as molecular recognition units enabling specific biological targeting. Moreover, the chemical flexibility of both BODIPY core and carbohydrate structures opens new avenues for designing multifunctional probes for specific applications, *e.g.* targeted imaging and therapeutic interventions. Accordingly, the mutual benefit of this integration resulted in fluorescent glycoconjugates that served in bioimaging applications to provide key insights into the trafficking of glycans^1–5^ or related lectins. Likewise, saccharides were included as structural recognition elements of the probe for targeting specific lectins in various immune and cancer settings.^1,2,6–9^

The clustering of glycans within the glycocalyx has inspired glycoscientists to develop structurally diverse scaffolds that can provide a multivalent display of sugar residues, thus improving the avidity of binding to lectins.^10–20^ Recently, some BODIPY cores served as scaffolds for the conjugation of multiple copies of sugar heads, resulting in improved lectin-mediated cell uptake by means of multivalent binding interactions with biological targets.^1^ The availability of an affordable and high fluorescent BODIPY with a core that can be easily functionalized is crucial for the development of advanced and multifunctional BODIPY-based architectures that can provide a multivalent display of sugar heads to a single BODIPY core.

**Fig. 1:**
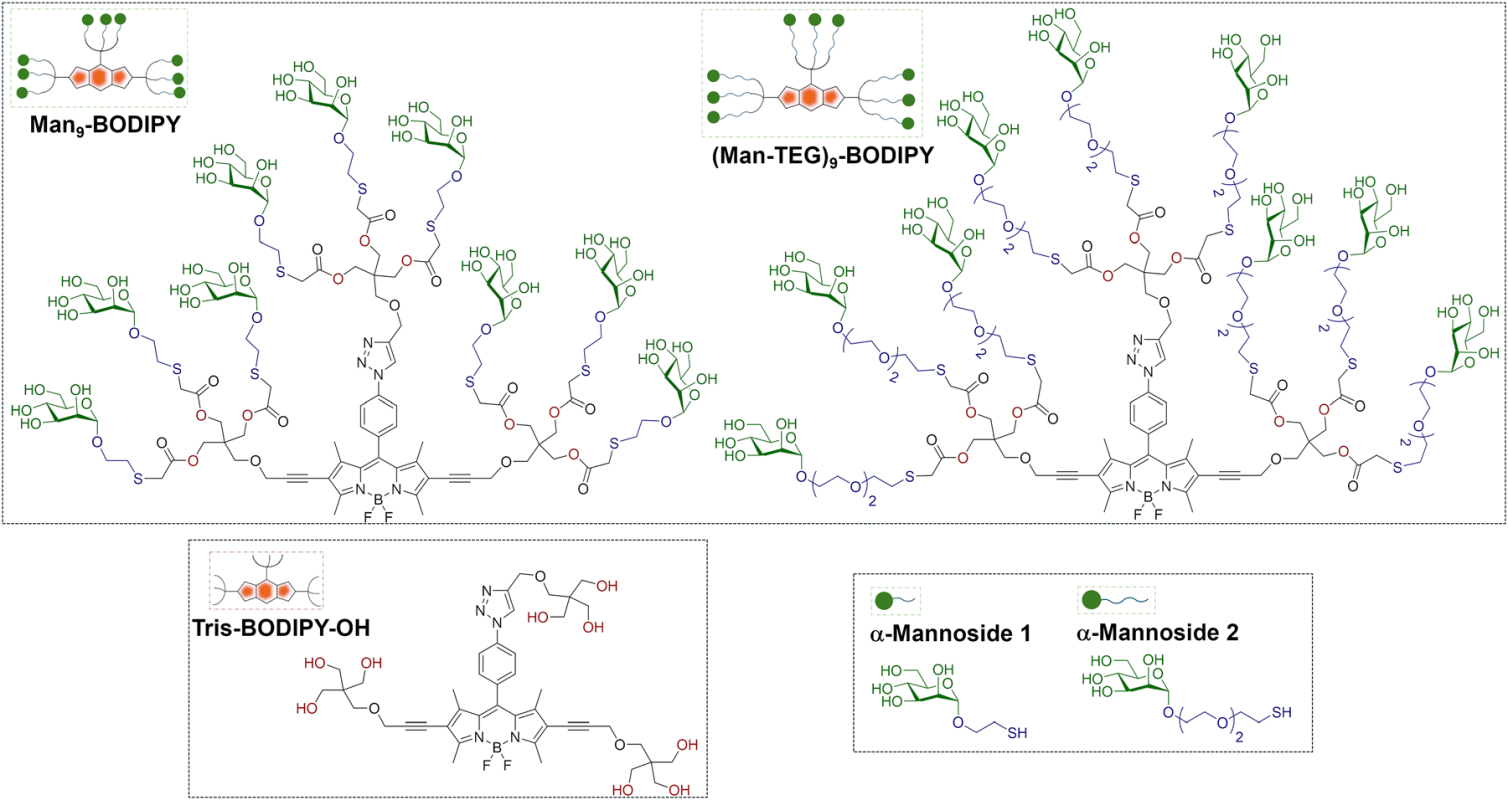
Structures and schematic representations of Tris-BODIPY-OH, α-mannosides 1 and 2 used in this work, Man_9_-BODIPY and (Man-TEG)_9_-BODIPY.

In this context, we have recently described the synthesis of the water-soluble Tris-BODIPY-OH (Fig. 1)^21^ that contains nine hydroxyl groups at the edges of the BODIPY core, and we demonstrated that it is a biocompatible probe for both *in vitro* and *in vivo* applications. In this work, we decided to investigate its potential as a multivalent and fluorescent platform for the display of multiple copies of sugars. Accordingly, we set out to explore the integration of sugar heads into Tris-BODIPY-OH, aiming to develop a multifunctional and modular molecular tool. In particular, d-mannose was selected as the sample sugar head for our study. Moreover, the intrinsic optical properties of Tris-BODIPY-OH facilitate the study of the sugar head arrangement on the BODIPY core and thus the biomolecular recognition with lectins using comparative fluorescence-based assays.

Tris-BODIPY-OH was combined with two suitable functionalized α-mannosides (compounds 1 and 2,^22,23^Fig. 1) which differ in the length of the linker at the anomeric position of the mannose. It resulted in two mannosylated BODIPYs, named Man_9_-BODIPY and (Man-TEG)_9_-BODIPY (Fig. 1), which display nine mannose residues conjugated to the BODIPY scaffold. Accordingly, we report here the design and synthesis of Man_9_-BODIPY and (Man-TEG)_9_-BODIPY, and a preliminary screening using the plant mannose-binding lectin concanavalin A (Con A). Then, the investigation of the binding of Man_9_-BODIPY to two relevant human lectins, *i.e.* dendritic cell-specific intercellular adhesion molecule-3-grabbing non-integrin (DC-SIGN) and langerin, was further performed.

Thanks to the intrinsic fluorescence properties of the core, Man_9_-BODIPY proved to be an efficient tool and facilitated the study of carbohydrate–lectin interactions using different and comparative fluorescence-based assays *i.e.* fluorescence lifetime imaging microscopy (FLIM), fluorescence polarization (FP) assay, and fluorescence correlation spectroscopy (FCS).

## Results and discussion

### Synthesis, optical characterization and agglutination studies of Man_9_-BODIPY and (Man-TEG)_9_-BODIPY

**Scheme 1:**
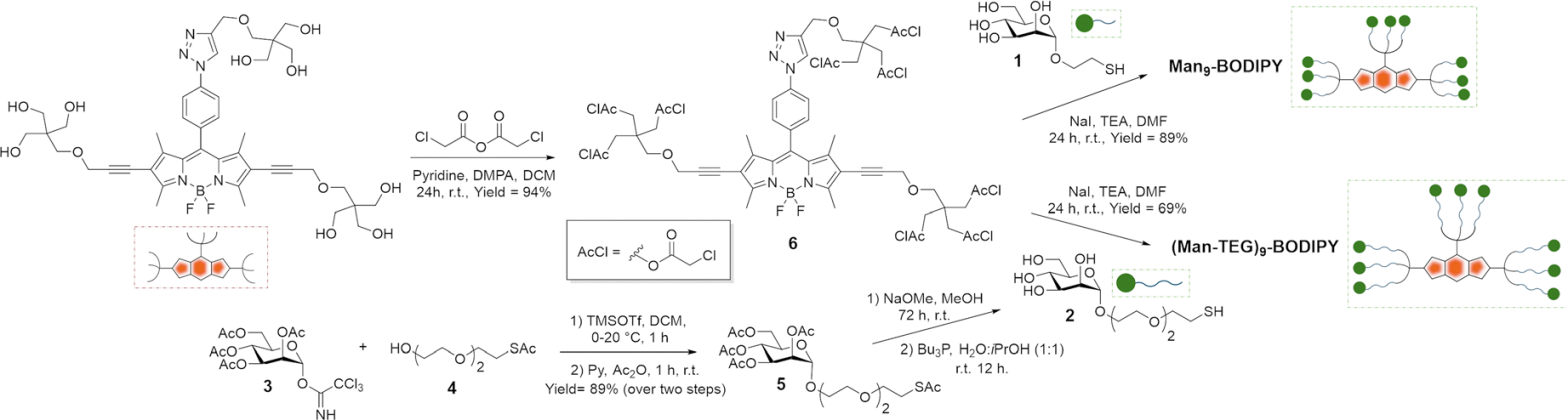
Synthesis of Man_9_-BODIPY and (Man-TEG)_9_-BODIPY.

The chloroacetyl thioether (ClAc) ligation^24,25^ was selected as a synthetic strategy for the assembly of Man_9_-BODIPY and (Man-TEG)_9_-BODIPY. In particular, Tris-BODIPY-OH (Scheme 1) and the α-mannoside 1, bearing a thiol-ending spacer at the anomeric carbon, were synthesized according to previously reported protocols.^21–23^ Then, the α-mannoside 1 was used as a crude mixture in the coupling reaction (see SI). The α-mannoside 2 bearing, at the anomeric carbon, a tetraethylene glycol (TEG) spacer with a terminal thioacetate group was prepared using the imidate 3^26^ (Scheme 1), as the glycosyl donor, and the acceptor 4^27^ in a glycosidation reaction with trimethylsilyl trifluoromethanesulfonate (see SI) to afford the protected derivative 5. Then, the hydrolysis of acetate groups under basic conditions, and the subsequent treatment of the crude with tributylphosphine afforded the α-mannoside 2, which was used as a crude mixture in the coupling reaction (see SI).

**Fig. 2:**
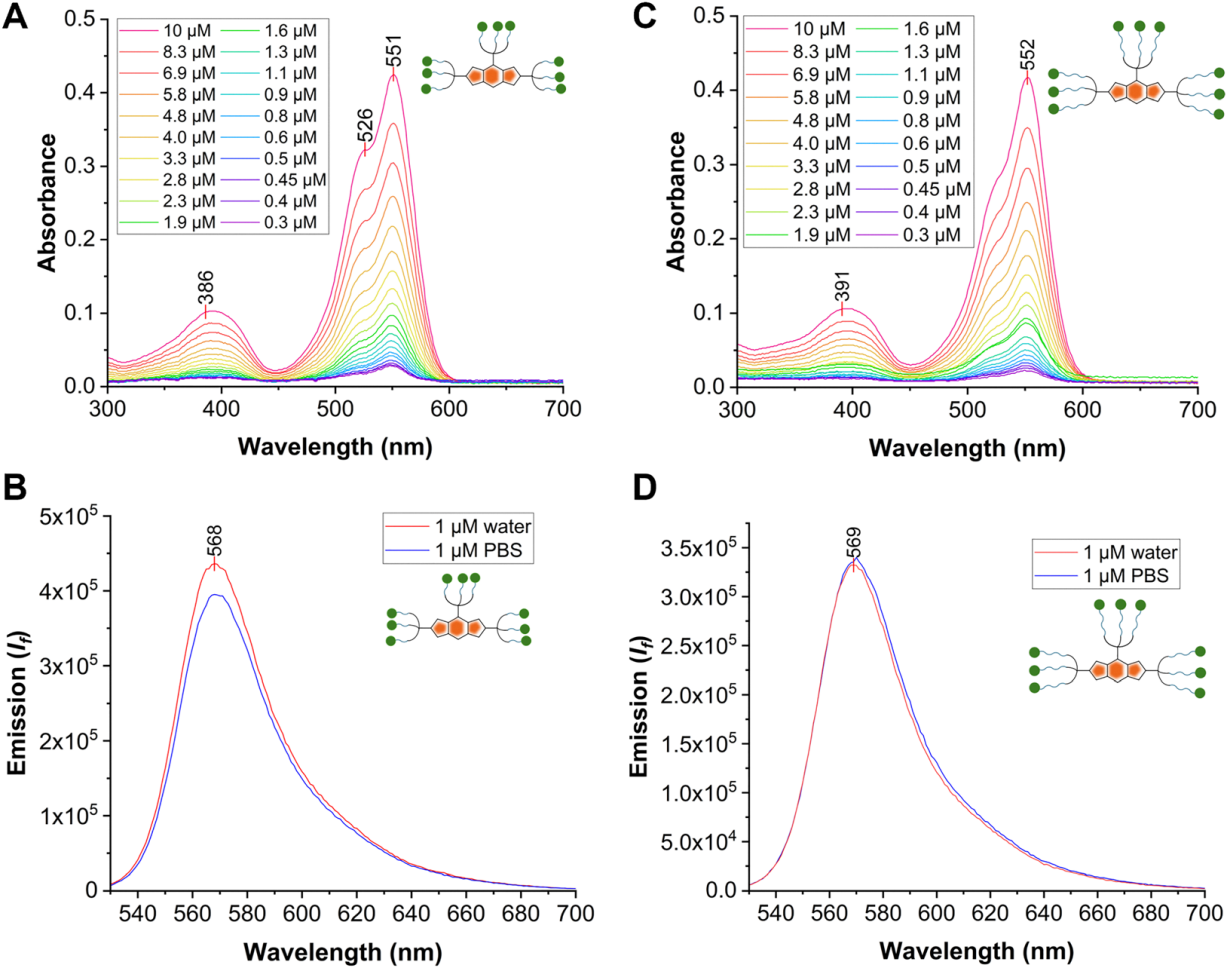
(A) Absorbance spectra of Man_9_-BODIPY in water at different concentrations (0.3–10 µM); (B) emission spectra of Man_9_-BODIPY (1 µM in water, and PBS pH = 7.4) after excitation at 520 nm; (C) absorbance spectra of (Man-TEG)_9_-BODIPY in water at different concentrations (0.3–10 µM); (D) emission spectra of (Man-TEG)_9_-BODIPY (1 µM in water, and PBS pH = 7.4) after excitation at 520 nm.

The nine hydroxyl groups of Tris-BODIPY-OH reacted with chloroacetic anhydride to afford compound 6 in high yield (94%). The chloroacetyl thiol ligation reactions between BODIPY **6** and the α-mannosides 1 and 2 were performed in dry *N*,*N*-dimethylformamide (DMF) in the presence of an excess of sodium iodide (NaI). The nonavalent glycoBODIPYs Man_9_-BODIPY and (Man-TEG)_9_-BODIPY were purified by subsequent trituration protocols with organic solvents (see the Experimental section), and then dialyzed *vs.* water (membrane cut-off 1000 Da). Accordingly, Man_9_-BODIPY and (Man-TEG)_9_-BODIPY were isolated in good yield (89% and 69% respectively) and analyzed by HPLC-MS and NMR spectroscopy (see SI, Fig. S1–S10). Next, the photochemical properties of Man_9_-BODIPY and (Man-TEG)_9_-BODIPY were evaluated (Fig. 2 and see SI Fig. S11 and S12).

In particular, the UV-vis absorption spectra were recorded in water and showed a similar absorption pattern previously reported for Tris-BODIPY-OH,^21^ with main absorption peaks around *λ* = 551 nm (see SI, Fig. 2A and C) assigned to the transition S0 → S1, and high molar extinction coefficients (4.1 × 10^4^ M^−1^ cm^−1^, see SI, Fig. S11 and S12). Upon excitation at 520 nm, emission spectra in both PBS and water showed emission peaks centered around 568 nm (see SI, Fig. 2B and D).

**Fig. 3:**
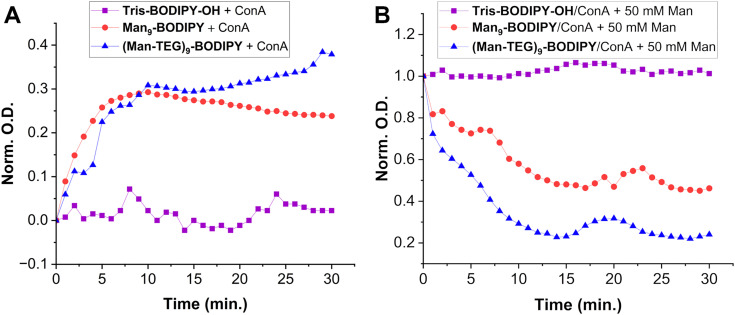
Turbidimetry analysis. (A) absorbance (*λ* = 490 nm) changes over time of a solution (20 µM) of Tris-BODIPY-OH (purple line), Man_9_-BODIPY (red line), and (Man-TEG)_9_-BODIPY (blue line) treated with a solution of ConA (20 µM in HEPES 25 mM, pH = 7,6, 1 mM CaCl_2_, 1 mM MnCl_2_) and (B) deagglutination over time of glycoBODIPYs/ConA by treatment with a solution of α-*O*-methyl-d-mannopyranoside (50 mM). Data are normalized according to the maximum optical density value.

Concanavalin A (ConA) was selected as a model of mannose-binding lectin^18^ to study if the arrangement of the mannose residues allowed for proper recognition by lectins’ carbohydrate binding domain. Accordingly, preliminary agglutination experiments were performed by following a previously reported protocol,^28^ thus expecting aggregation of lectin with the mannosylated BODIPYs driven by the molecular recognition of the mannose residues on the fluorescent probes. Accordingly, Tris-BODIPY-OH was used as a negative control. The changes in the absorption (*λ* = 490 nm) of the solutions of the glycoBODIPYs treated with a fixed concentration of ConA (20 µM in HEPES 25 mM, pH 7.6, 1 mM CaCl_2_, 1 mM MnCl_2_) were monitored over time (0–30 min, Fig. 3A).

Data obtained indicated that the turbidity of the solutions of both glycoBODIPYs increased over time (Fig. 3A, blue and red lines). Moreover, the addition of a solution of α-*O*-methyl-d-mannopyranoside (50 mM) resulted in a fast deagglutination process with a decrease in the turbidity of the solutions (Fig. 3B, blue and red lines). No change in the absorption was observed in the Tris-BODIPY-OH/ConA mixture (Fig. 3A, purple line). Altogether, these data confirmed that aggregation was a consequence of the recognition of the mannose residues on the BODIPYs.

### Man_9_-BODIPY is a useful probe in fluorescence-based assays: fluorescence lifetime imaging microscopy (FLIM), fluorescent polarization (FP) assay and fluorescent correlation spectroscopy (FCS)

**Fig. 4:**
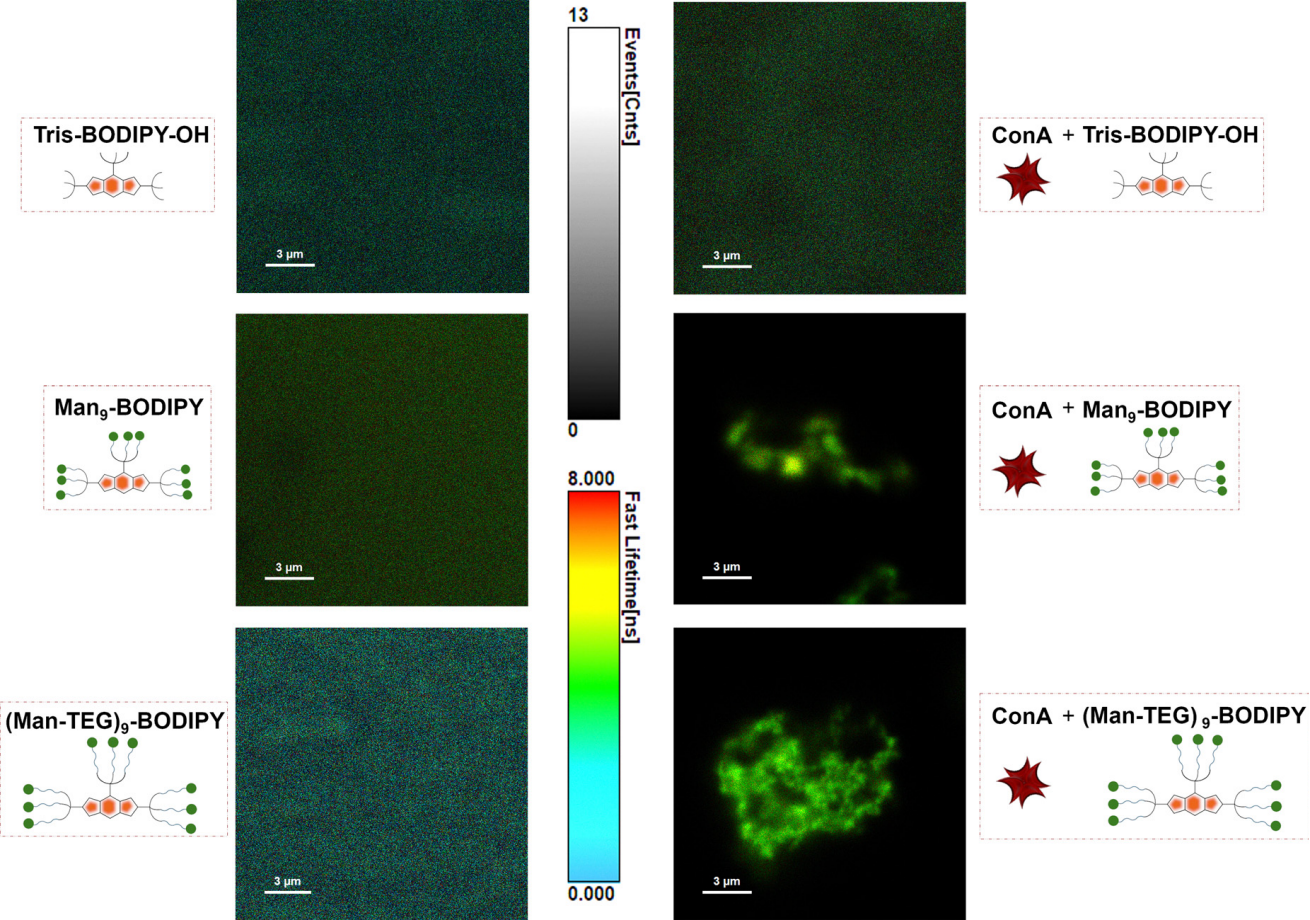
FLIM images measured for: (left panels) the (free) BODIPY solutions 500 nM in HEPES 25 mM pH 7.6, 1 mM CaCl_2_, 1 mM MnCl_2_ and for (right panels) solutions of the BODIPYs (500 nM) combined with ConA (500 nM in HEPES 25 mM pH 7.6, 1 mM CaCl_2_, 1 mM MnCl_2_). The gray scale-bar represents the number of photon events counted for a pixel, while the color scale-bar represents the measured lifetime.

Fluorescence lifetime imaging microscopy (FLIM)^29^ is a widely applied and impactful technique capable of revealing changes in the molecular environment of fluorescent dyes. These changes are not evident in other spectral techniques and are detected by measuring the lifetime of a fluorophore in an excited state before it decays to a less energetic state by emitting a photon. FLIM has been successfully applied to investigate intermolecular interactions, protein conformations and concentrations of analytes, among other things. Accordingly, FLIM was used in this work to directly visualize the formation of the glycoBODIPY/ConA complexes. Fig. 4 shows FLIM images of the (free) BODIPY solutions (left panels) and in the presence of ConA (right panels). The color scale in the images corresponds to the dye lifetime measured by FLIM for each pixel in the image. As expected, the dye distribution and the lifetime distribution are essentially uniform in the free dyes. One can notice a slight shift in the color of the Man_9_-BODIPY towards green/yellow, indicating a longer lifetime. This is confirmed by the histograms of the detected lifetimes (see SI, Fig. S13) for all three BODIPYs. In the presence of ConA, a clear difference is observed between Tris-BODIPY-OH and the mannosylated BODIPYs. While the distributions of dye and its lifetime remain uniform for Tris-BODIPY-OH, we observe the presence of ConA aggregates for the mannosylated BODIPYs and its fluorescence lifetimes clearly shifted towards green/yellow, *i.e.* longer lifetimes. This finding is again quantitatively confirmed by the lifetime histograms (see SI, Fig. S13): for Tris-BODIPY-OH, the peak of the histogram is unchanged and one observes only a broadening, for the mannosylated BODIPYs the entire histogram is shifted to longer lifetimes, as an effect of the formation of the complex. It should be noted that the lifetime histograms for Man_9_-BODIPY and (Man-TEG)_9_-BODIPY are essentially comparable in the presence of ConA (see SI, Fig. S13).

**Fig. 5:**
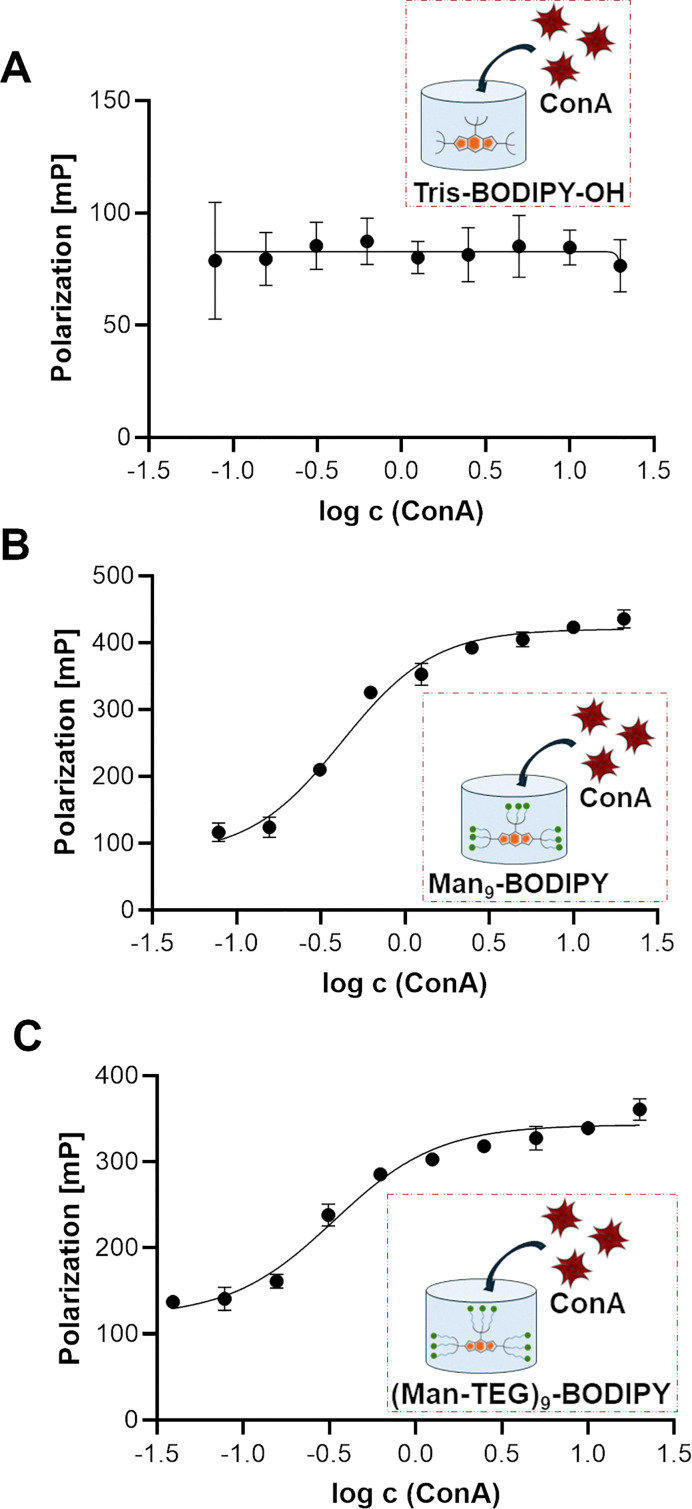
Binding of ConA to Tris-BODIPY-OH (A), Man_9_-BODIPY (B), and (Man-TEG)_9_-BODIPY (C). A fixed concentration of the fluorescent probes (0.5 µM) was mixed with a range of increasing concentrations of Con A (*X*-axis), and fluorescence polarization was measured (*Y*-axis). The following settings were used for the FP assay: *λ*_ex_ =520/15 nm and *λ*_em_ = 555/15 nm, calibrated G-factor for BODIPYs. All experiments were conducted at 25 °C.

Fluorescence polarization (FP) is a widely used technique in biochemical and medicinal chemistry research for studying ligand–protein interactions, protein–protein interactions and antigen–antibody interactions. The major advantage of FP over similar methods is that it enables real-time measurements in solution, it does not require the manipulation of the target protein (*e.g*. immobilization to a chip or on microtiter plate), making it an excellent method for screening compound libraries in a high-throughput protocol.^30^ One of the most critical aspects of the FP assay is the requirement of a fluorescent probe with both high quantum yield and high photostability,^30^ properties that both glycoBODIPYs, Man_9_-BODIPY and (Man-TEG)_9_-BODIPY, exhibit. Furthermore, the probe should exhibit selective binding based on the “ligand” part rather than the fluorescent moiety. Accordingly, the binding properties of Man_9_-BODIPY and (Man-TEG)_9_-BODIPY with ConA were evaluated in a FP assay.^18^Tris-BODIPY-OH was used as a negative control to confirm that the fluorescent moiety alone does not promote nonspecific binding. In three separate experiments, a fixed concentration of BODIPYs was incubated with increasing concentrations of ConA. As expected, Tris-BODIPY-OH did not bind to ConA (Fig. 5A, see SI Fig. S14A).

Conversely, a dose-dependent increase of fluorescence polarization was observed in the titration experiments of ConA with Man_9_-BODIPY (Fig. 5B, see SI Fig. S14B) and with (Man-TEG)_9_-BODIPY (Fig. 5C, see SI Fig. S14C). The experiment also showed a high binding affinity for both mannosylated BODIPYs, which is due to the multivalent display of the mannose heads on the BODIPY core. *K*_d_ values in the nanomolar range (see SI, Table S1, 300 nM for Man_9_-BODIPY and 120 nM for (Man-TEG)_9_-BODIPY) were observed for the binding of glycoBODIPYs to ConA. Altogether, these data indicated that the length of the linker had a minor and barely significant effect on the recognition of the mannose residues by the carbohydrate recognition domains of lectin. Accordingly, looking at the differences observed in the lifetime of the two glycoBODIPY probes in their unbound (free) state (see SI Fig. S13), we decided to proceed further with the investigations with Man_9_-BODIPY, which is the probe that showed the highest lifetime values and longer-lived excited states may indicate more photostable or less environmentally sensitive fluorophores.

**Fig. 6:**
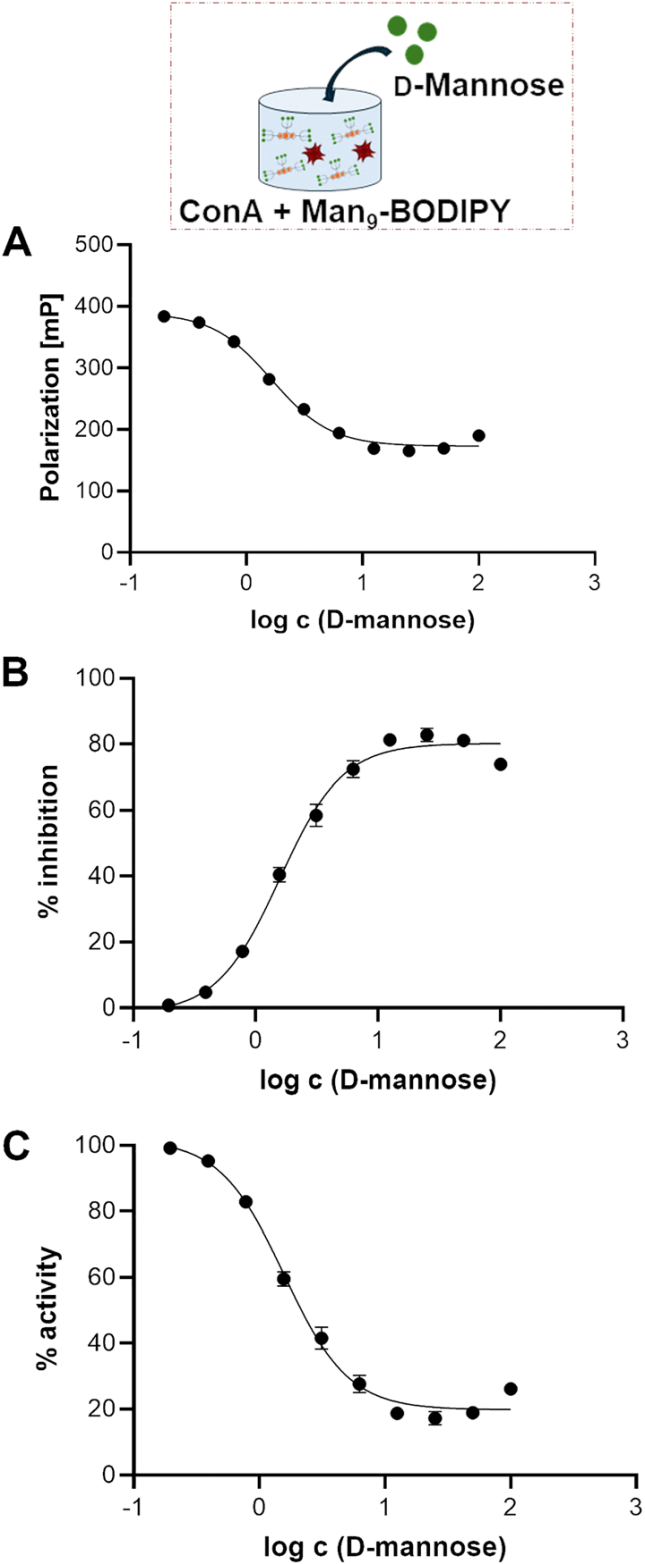
Binding curves (A–C) of the competitive FP assays using fixed concentrations of Man_9_-BODIPY (0.5 µM) and ConA (2 µM) and adding increasing concentrations of d-mannose. The fluorescence polarization is expressed in millipolarization (mP) units (A). The relationship between relative inhibition (%, B), activity (%, C) and log c (d-mannose) is also shown. Relative inhibition refers to competitive inhibition of Man_9_-BODIPY binding to ConA normalised to the maximum absolute inhibition achieved, while relative activity refers to the values describing relative Man_9_-BODIPY binding to ConA.

Titration experiments allowed us to define the concentration of ConA to be used in a competitive displacement assay, which was set to 2 µM. To further evaluate the possibility to use this glycoBODIPY probe in a competitive test, we investigated the ability of Man_9_-BODIPY to be efficiently displaced by a potential lectin-binding ligand. Accordingly, Man_9_-BODIPY was used in a competitive setting with d-mannose as the lectin-binding ligand (Fig. 6).

A solution of Con A was thus treated with a fixed concentration of Man_9_-BODIPY and then changes in the fluorescence polarization according to the addition of a range of increasing concentrations of d-mannose were analysed. Binding curves and binding properties of d-mannose are shown in Fig. 6 (see also SI, Fig. S15). The addition of d-mannose resulted in a dose-dependent reduction of the observed fluorescence polarization, proving that d-mannose competitively displaced the probe (Fig. 6A). Accordingly, the *K*_d_ value of d-mannose resulted in 1.35 mM (see SI, Table S2), which is in line with previously reported values.^31,32^

**Fig. 7:**
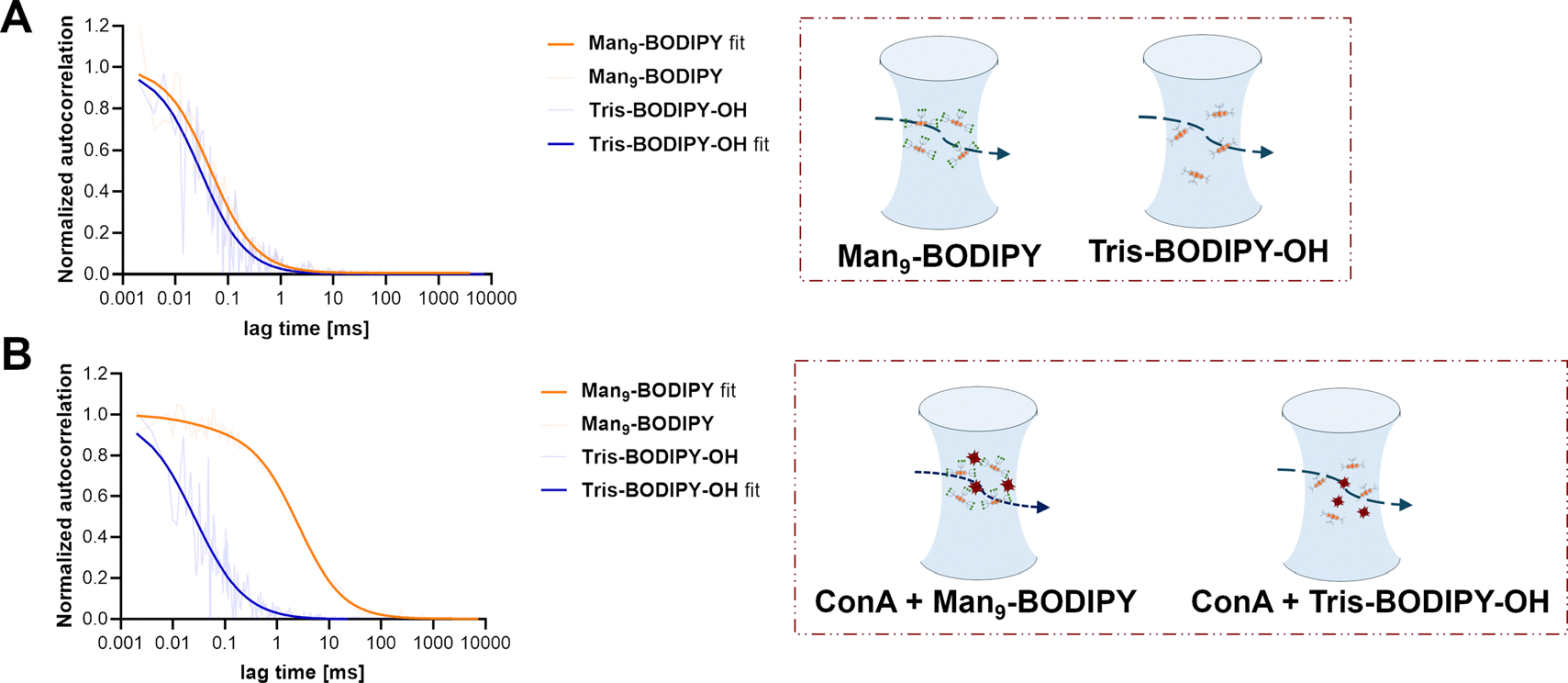
Fluorescence autocorrelation curves for both probes (Man_9_-BODIPY and Tris-BODIPY-OH, colours as indicated in the legend) in the absence (A) and presence (B) of ConA, where the right-shifted curve for Man_9_-BODIPY (orange) indicates a slowdown in diffusion of the probe due to binding to the protein. The light thin lines represent the correlated data, while the smooth curves correspond to the fits by a 3D-diffusion model.

Then, fluorescence correlation spectroscopy (FCS) was used as a comparative technique to further support our findings. FCS reports on differences in translational diffusion^33,34^ rather than rotational diffusion in the polarization experiments described above. Accordingly, the binding of Tris-BODIPY-OH and Man_9_-BODIPY to ConA was evaluated. Fig. 7 shows the fluorescence autocorrelation curves for both probes. Without the protein (ConA) a slight shift in the curve towards longer lag times was observed for Man_9_-BODIPY, as a consequence of its slower diffusion compared to that of Tris-BODIPY-OH (Fig. 7A).

This is in line with the higher molecular weight of the mannosylated BODIPY compared to its non-glycosylated precursor Tris-BODIPY-OH. In the presence of ConA, the pronounced right-shift of the curve for Man_9_-BODIPY indicates an approximately 50-fold slowdown of the diffusion rate (Fig. 7B and see SI, Fig. S16), which independently confirms the probe's multivalent binding to ConA. Monovalent attachment of a roughly 2 kDa probe to a 100 kDa globular protein should result in a slowdown of approx. 4-fold. Conversely, no shift was observed when Tris-BODIPY-OH was exposed to ConA.

To further validate the robustness of our study and the potential for studying the carbohydrate–lectin interactions of Man_9_-BODIPY with other lectins, we have included two relevant human lectins in this study. The molecular and immunological cross-talk between the host and the environment (from pathogens to the tumour microenvironment) is primarily driven by carbohydrate-mediated interactions. Most of the antigen-presenting cells are equipped with a library of pattern recognition receptors that can sense and recognize damage-associated molecular patterns. Among the pattern recognition receptors, lectins are conserved receptors able to modulate innate immune responses. In particular, DC-SIGN and langerin are two important C-type lectins involved in several immunomodulatory phenomena.^35,36^ These lectins recognize mannose residues, which is why they were selected as the model human lectins for this study.

**Fig. 8:**
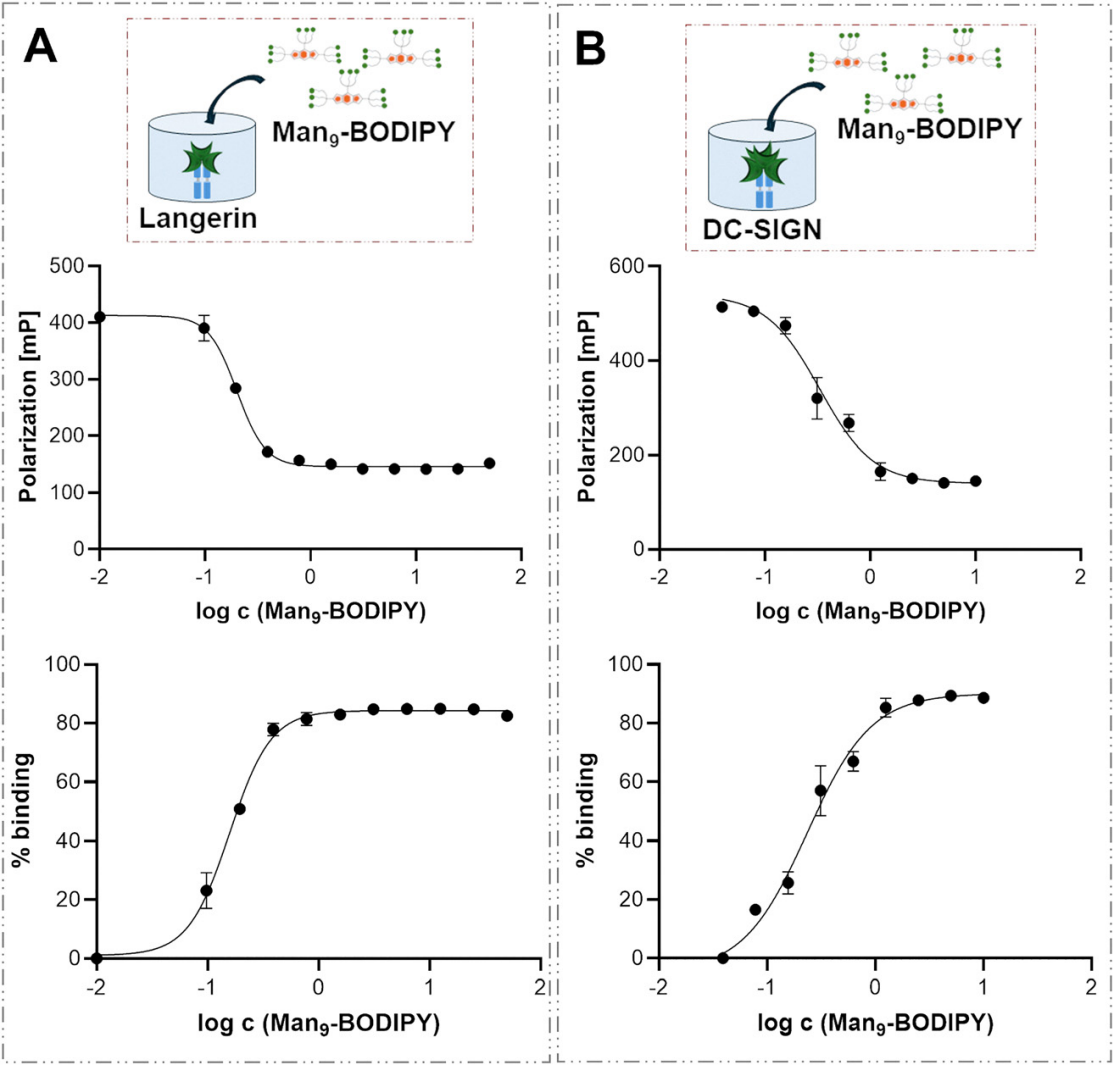
(A) Binding of langerin to Man_9_-BODIPY. A fixed concentration of langerin (0.5 µM) was mixed with a range of concentrations of Man_9_-BODIPY (*X*-axis), and fluorescence polarization was measured (*Y*-axis). (B) Binding of DC-SIGN to Man_9_-BODIPY. A fixed concentration of DC-SIGN (0.05 µM) was mixed with a range of concentrations of Man_9_-BODIPY (*X*-axis), and fluorescence polarization was measured (*Y*-axis).

DC-SIGN and langerin chimera lectins^37,38^ were used in this study. Due to the lower availability of these lectins compared to ConA, titrations were performed using fixed concentrations of langerin (Fig. 8A) or DC-SIGN (Fig. 8B) lectins, with increasing concentrations of the Man_9_-BODIPY added in two separate titration experiments (Fig. 8, Table 1, and see SI Fig. S17).

**Table 1:** Binding properties of Man_9_-BODIPY against Con A, langerin and DC-SIGN

	Con A	Langerin	DC-SIGN
*K*_d_ [µM]	0.30 ± 0.04	0.20 ± 0.01	0.33 ± 0.08
m*P*_max_ [mP]	439 ± 27	413 ± 14	541 ± 54
m*P*_0_ [mP]	93.3 ± 12.9	73.7 ± 2.8	76.7 ± 6.2

The interaction between the mannose residues of Man_9_-BODIPY and the target lectins was successfully analyzed using the FP assay. In this case, the binding curve is inverted compared to that in Fig. 8B, as increasing the probe concentration leads to a higher fraction of free probe, which depolarizes the light. Specifically, Man_9_-BODIPY displayed binding affinities (*K*_d_ values) in the nanomolar range for both C-type lectins, 330 nM for DC-SIGN and 200 nM for langerin. Overall, these data demonstrate that Man_9_-BODIPY arranges the sugar heads on the BODIPY core in a way that facilitates the recognition by the carbohydrate-binding domains of the three different lectins.

## Conclusions

We report here the use of the BODIPY dye as a scaffold for the multivalent display of sugar heads. The resulting Man_9_-BODIPY exhibited a nanomolar affinity towards the studied lectins due to the clustering of the sugar heads on the BODIPY core, proving to be a valuable tool for studying carbohydrate–lectin interactions in comparative fluorescence-based assays. Notably, glycosides containing a thiol-ending spacer at the anomeric position are readily available. Using the chloroacetyl thioether ligation reaction as a synthetic strategy to conjugate nine copies of α-mannosides demonstrates the versatility of this approach, which was used to prepare two glycoBODIPYs with different linkers. This makes Tris-BODIPY-OH a universal scaffold for conjugating other sugar heads or glycomimetics, thus expanding the range of applications of the resulting glycosylated BODIPY probes. Combining the multivalent presentation of sugar heads with BODIPY's intrinsic fluorescence properties enables the development of glycosylated fluorescent tools with an improved affinity for target lectins.^39^ These tools could be useful for studying lectin trafficking in *in vitro* or *in vivo* settings, or for bioimaging and therapeutic applications involving pathogen-derived biofilms.^40^

## Experimental

### Materials and methods

All reagents, whose synthesis is not described, were commercially available and were used without any further purification, if not specified otherwise. ConA was purchased from Sigma Aldrich (L7647-100MG). NMR spectra were recorded on Varian Inova 400, Mercury plus 400 and Gemini 200 instruments. Chemical shifts were reported in parts per million (ppm) relative to the residual solvent peak, rounded to the nearest 0.01 for proton and 0.1 for carbon (reference: CHCl_3_ [^1^H: 7.26 ppm, ^13^C: 77.0 ppm]). Coupling constants *J* were reported in Hz to the nearest 0.01 Hz. Peak multiplicity was indicated as follows s (singlet), d (doublet), t (triplet), q (quartet), m (multiplet), br (broad signal) ad (apparent doublet) and aq (apparent quartet). ESI-MS were recorded on an LC-MS LCQ Fleet ThermoFisher Scientific system. UV-vis spectra were recorded on BMG Labtech Spectrostar Nano using a 1 cm quartz cell or a 96 well plate. Fluorescence spectra were recorded on a HORIBA FluoroMax Plus spectrofluorimeter using a 1.0 cm cell. Flash chromatography was performed on Merck silica gel 60 (0.040–0.063 mm). The thin layer chromatography was performed on a Supelco TLC silica gel 60 F254 system (aluminum sheets or glass plates). High resolution mass analyses were performed with a resolution of 70000 FWHM at *m*/*z* = 200 in an alternate electrospray mode with data-dependent acquisition of HCD fragmentation spectra (resolution 17500 FWHM at *m*/*z* = 200) of the more abundant monocharged ions (Q-Exactive hybrid quadrupole – orbitrap mass analyzer, Thermo Scientific).

### Synthesis of 6

Tris-BODIPY-OH (23 mg, 0.026 mmol) was suspended in dichloromethane (1.15 mL), then pyridine (28 µL, 0.35 mmol), chloroacetic anhydride (60 mg, 0.35 mmol) and DMAP (0.95 mg, 7.8 × 10^−3^ mmol) were added. The reaction mixture was stirred at r.t. for 24 h, then it was diluted with dichloromethane (100 mL) and washed with a saturated solution of ammonium chloride (3 × 10 mL), and with brine (1 × 10 mL). The organic phase was dried with Na_2_SO_4_, filtered, and concentrated under vacuum to give 6 (38 mg, 94%) as a reddish glassy solid. ^1^H-NMR (400 MHz, CDCl_3_) *δ*: 8.12 (s, 1H), 8.00–7.99 (m, 2H), 7.49–7.47 (m, 2H), 4.72 (s, 2H), 4.35 (s, 4H), 4.28 (s, 6H), 4.25 (s, 12H), 4.06 (s, 6H), 4.03 (s, 12H), 3.63 (s, 2H), 3.57 (s, 4H), 2.64 (s, 6H,), 1.52 (s, 6H).^13^C-NMR (101 MHz, CDCl_3_) *δ*: 166.8, 166.8, 159.2, 145.5, 144.7, 140.8, 137.7, 134.7, 130.8, 129.6, 121.2, 120.9, 115.6, 91.6, 78.8, 68.0, 67.4, 64.6, 64.0, 63.8, 59.6, 43.2, 43.0, 40.7, 40.6, 13.8. HR-MS: calcd. for C_61_H_65_BCl_9_F_2_N_5_NaO_21_ [M+Na]+ 1594.1263 found 1594.1173 Δ = −5.61 ppm.

### Synthesis of Man_9_-BODIPY

In a Schlenk flask, 6 (17.3 mg, 0.011 mmol) was dissolved in dry *N*,*N*-dimethylformamide (0.5 mL), then sodium iodide (33 mg, 0.22 mmol) was added, and the mixture was degassed with three cycles of vacuum–argon. In a second flask, 1 (36 mg, 0.15 mmol) was dissolved in dry *N*,*N*-dimethylformamide (0.5 mL) and degassed with three cycles of vacuum–argon. Then, the solution of α-mannoside 1 and dry DIPEA (105 µL, 0.605 mmol) was added to the solution of BODIPY 6. The reaction mixture was stirred at r.t. The reaction progression was monitored by C18 modified silica thin layer chromatography (acetonitrile : water 1 : 1). After the complete consumption of BODIPY 6 (19 h), the solvent was removed under vacuum and the solid was washed with Et_2_O (5 × 2 mL) and DCM (5 × 2 mL). Then the crude was dissolved in milliQ water (2 mL) and dialyzed *vs.* water (MWCO 1000 Da, 1L milliQ water, replaced every 8 h) for 24 h. Then the solution was freeze-dried affording Man_9_-BODIPY (33.6 mg, 89%) as a highly hygroscopic fluffy solid.^1^H-NMR (400 MHz, D_2_O) *δ*: 8.37 (bs, 1H), 7.82 (bs, 2H), 7.13 (bs, 2H), 4.77–4.66 (m, 11H), 4.22 (bs, 4H), 4.03 (bs, 17H), 3.82–3.37 (m, 76H), 3.24–3.19 (m, 9H), 2.65 (d, *J* = 17.1 Hz, 16H), 2.33 (bs, 6H), 1.27 (bs, 6H). ^13^C-NMR (101 MHz, D_2_O, 60 °C) *δ*: 171.7, 171.5, 146.1, 144.9, 141.6, 137.9, 134.4, 131.0, 122.2, 121.3, 100.3, 100.2, 93.5, 78.8, 73.3, 71.2, 70.5, 67.1, 66.8, 64.0, 61.3, 43.5, 43.3, 33.9, 32.2, 13.8. HR-MS: calcd for C_133_H_202_BF_2_N_5_O_75_S_9_ [M+2H]^2+^ 1703.9864 found 1703.9869 *Δ* = −0.24 ppm.

### Synthesis of (Man-TEG)_9_-BODIPY

In a Schlenk flask, 6 (23.2 mg, 0.015 mmol) was dissolved in dry *N*,*N*-dimethylformamide (0.75 mL), then sodium iodide (45 mg, 0.3 mmol) was added and the mixture was degassed with three cycles of vacuum–argon. In a second flask, α-mannoside 2 (65.8 mg, 0.2 mmol) was dissolved in dry *N*,*N*-dimethylformamide (0.75 mL) and degassed with three cycles of vacuum–argon. Then, the solution of 2 and dry DIPEA (143 µL, 0.825 mmol) was added to the solution of BODIPY 6. The reaction mixture was stirred at r.t. The reaction progression was monitored by C18 modified silica thin layer chromatography (acetonitrile : water 1 : 1). After the complete consumption of BODIPY 6 (19 h), the solvent was removed under vacuum and the solid was washed with Et_2_O (5 × 2 mL) and dichloromethane (5 × 2 mL). Then the crude was dissolved in milliQ water (2 mL) and dialyzed *vs.* water (MWCO 1000 Da, 1 L milliQ water, replaced every 8 h) for 24 h. Then the solution was freeze-dried affording (Man-TEG)_9_-BODIPY (42.0 mg, 69%) as a highly hygroscopic, fluffy solid. ^1^H-NMR (400 MHz, D_2_O) *δ*: 8.50 (bs, 1H), 7.95 (bs, 2H), 7.31 (bs, 2H), 4.81 (bs, 16H), 4.14 (bs, 36H), 3.98–3.06 (m, 30H), 2.71 (s, 30H), 2.47 (s, 5H), 0.73 (s, 5H). ^13^C-NMR (101 MHz, D_2_O, 60 °C) *δ*: 171.7, 100.4, 100.3, 73.2, 71.1, 70.5, 70.5, 70.1, 70.0, 67.2, 66.8, 63.9, 61.4, 61.3, 43.3, 33.8, 32.1, 23.7, 23.2, carbons of the BODIPY were not visible under these conditions. HR-MS: calcd for C_169_H_274_BF_2_N_5_O_75_S_9_ [M+2H]^2+^ 2098.72188 found 2098.71997, *δ* = −0.91 ppm.

### C-type lectins-Fc

DC-SIGN lectin used in this work is DC-SIGN-Fc that consists of the extracellular portion of DC-SIGN fused to a human IgG1-Fc fragment.^37^ Langerin lectin used in this work is Langerin-Fc which was generated by amplifying RNA encoding the extracellular domains of langerin and fused to human IgG1-Fc.^38^

### Turbidimetry analysis

The agglutination assay was carried out as reported in the literature with minor modifications.^28^Tris-BODIPY-OH, Man_9_-BODIPY and (Man-TEG)_9_-BODIPY were first dissolved in dimethyl sulfoxide (1 mM), then the BODIPY solutions were diluted to 20 µM in the buffer (HEPES 25 mM pH 7.6, 1 mM CaCl_2_, 1 mM MnCl_2_). A solution of ConA (20 µM) in the buffer (HEPES 25 mM pH 7.6, 1 mM CaCl_2_, 1 mM MnCl_2_) was prepared. In a 96-well plate (Microplate, 96 wells, PS, F-BOTTOM (CHIMNEY WELL), clear, non-binding) were placed 100 µL of the 20 µM solution of each BODIPY, then 100 µL of the solution of ConA was added. The mixture was gently shaken for 10 minutes, then the optical density at *λ* = 490 nm was measured every minute for 30 minutes. The measurements were carried out in triplicate. Deagglutination was performed by adding a solution of α-*O*-methyl-d-mannopyranoside directly to the 96-well plate to reach 50 mM concentration in the well and monitoring the decrease in the optical density at *λ* = 490 nm.

### Fluorescence lifetime imaging (FLIM)

FLIM was performed at room temperature (20 °C) on a Nikon Ti AX-R NSPARC confocal microscope using a Nikon Plan Apo *λ*D 100× oil immersion objective (NA = 1.45). The microscope is equipped with a two-channel PicoQuant FLIM compact module system. Images of 1024 × 1024 pixels were acquired using a 488 nm pulsed laser for excitation and recording photons of fluorescence emission at a wavelength of 620 ± 50 nm for 90 s at a frequency of 25 MHz through a PMA Hybrid single photon counting module and a MultiHarp 150 multichannel event timer. Lifetime histograms were calculated and analyzed using SymphoTime©. Samples were inserted into LabteK chambers, and the images were acquired at 10 µm from the coverslip to avoid interactions of the dye with the surface of the coverslip. Sample preparation: 1 mM stock solutions of Tris-BODIPY-OH, Man_9_-BODIPY and (Man-TEG)_9_-BODIPY in pure dimethyl sulfoxide were prepared and later diluted to 500 nM in HEPES 25 mM, 1 mM CaCl_2_, 1 mM MnCl_2_ buffer (pH = 7.6); FLIM was performed on the 500 nM solution of each dye in the absence or presence of 500 nM Con A.

### Fluorescence polarization (FP)

Fluorescence polarization assays were performed using a Tecan Spark multimode microplate reader (Tecan Trading AG, Switzerland), where fluorescence polarization of BODIPY probes Tris-BODIPY-OH, Man_9_-BODIPY and (Man-TEG)_9_-BODIPY was measured by excitation at 520/15 nm and emission at 555/15 nm. 1 mM stock solutions of Tris-BODIPY-OH, Man_9_-BODIPY and (Man-TEG)_9_-BODIPY were prepared in pure dimethyl sulfoxide and later diluted in the buffer (HEPES 25 mM, 1 mM CaCl_2_, 1 mM MnCl_2_, pH = 7.6) to the final concentration of 1 µM. Concanavalin A (ConA) was dissolved in the same buffer and a 100 µM stock solution was prepared, which was later diluted to 40 µM. ConA (20–0.078 µM) was titrated against a fixed concentration of Tris-BODIPY-OH, Man_9_-BODIPY and (Man-TEG)_9_-BODIPY (0.5 µM). All experiments were performed at room temperature (25 °C).

### Competitive fluorescence polarization assay

Fixed concentrations of Man_9_-BODIPY (0.5 µM) and ConA (2 µM) were used in a competitive fluorescence polarisation assay. The highest d-mannose concentration was 100 mM and the lowest was 190 µM. All experiments were performed at room temperature (25 °C).

### Fluorescence correlation spectroscopy (FCS)

1 mM stock solutions of Tris-BODIPY-OH and Man_9_-BODIPY in pure dimethyl sulfoxide were prepared and later diluted in HEPES 25 mM, 1 mM CaCl_2_, and 1 mM MnCl_2_ buffer (pH = 7.6). 500 nM solution of each dye in the absence or presence of 50 nM ConA was placed in a glass-bottom ibidi 18-well µ-slides, precoated with albumin (Sigma Aldrich) to reduce dye adsorption to the surface. FCS experiments were performed with an Abberior Instruments microscope in the confocal mode using the Olympus UPlanSApo 60×/1.2 water immersion objective and steered through the Imspector software. Fluorescence was excited by a 561 nm pulsed excitation laser at an average power of around 10 µW, and detected by an avalanche photodiode through a 0.9 Airy-unit pinhole and a 580–630 nm emission filter (Semrock). Intensity fluctuations were acquired in 2 µs bins for 15–120 seconds at least 5 times per sample. The intensity traces were autocorrelated and further analysed with the open source FoCuS-scan software (https://github.com/dwaithe/FCS_scanning_correlator)^41^ employing the 3D-diffusion model (fits in Fig. 8) with the aspect ratio of the optical point-spread-function and anomalous diffusion exponent fixed to 10 and 0.9, respectively. The obtained transit times (Fig. S17A) were transformed into diffusion coefficients (Fig. S17B) according to the measurements of the reference probe rhodamine B with a known diffusion coefficient.^42^ Data for the samples with ConA were fit with a two-component model describing two diffusing species – the free probe (parameters fixed to the values obtained for that probe alone), and bound probe (fraction displayed in Fig. S17C).

## Author contributions

G. B. (data curation: lead; formal analysis: lead; investigation: lead; validation: lead; and visualization: lead); E. P. (data curation: lead; formal analysis: lead; investigation: lead; validation: lead; and visualization: lead); J. T. (data curation: equal; formal analysis: equal; investigation: equal; validation: equal; and visualization: equal); J. M. (data curation: equal; formal analysis: equal; investigation: equal; validation: equal; and visualization: equal); S. C. (funding acquisition: equal; methodology: supporting; visualization: supporting; and writing – review & editing: supporting); M. L. (methodology: equal; investigation: lead; resources: equal; and writing – review & editing: equal); Y. K. (methodology: supporting; resources: lead; and writing – review & editing: supporting); F. C. (methodology: equal; resources: equal; and writing – review & editing: supporting); I. U. (methodology: equal; resources: equal; supervision: lead; and writing – review & editing: equal); M. A. (funding acquisition: lead; methodology: lead; resources: lead; supervision: lead; visualization: equal; writing – original draft: lead; and writing – review & editing: lead); B. R. (conceptualization: lead; funding acquisition: lead; methodology: lead; project administration: lead; resources: lead; supervision: lead; visualization: lead; writing – original draft: lead; and writing – review & editing: lead).

## Conflicts of interest

There are no conflicts to declare.

## Abbreviations

Con A: Concanavalin A
DC-SIGN: Dendritic cell-specific intercellular adhesion molecule-3-grabbing non-integrin
FP: Fluorescence polarization
FCS: Fluorescence correlation spectroscopy
FLIM: Fluorescence lifetime imaging microscopy

## Supplementary Material

CB-OLF-D5CB00190K-s001

## Data Availability

The data supporting this article have been included as part of the supplementary information (SI). Supplementary information is available. See DOI: https://doi.org/10.1039/d5cb00190k.
