# Supplementary material for: A nonavalent BODIPY with a multivalent arrangement of α-mannosides enables lectins recognition in fluorescence-based assays
Source: RSC Chem Biol. 2025 Dec 5;7(3):444–54. doi: 10.1039/d5cb00190k (PMC12757955; doi:10.1039/d5cb00190k)
Supplement: CB-007-D5CB00190K-s001 [file CB-007-D5CB00190K-s001.pdf]

Supplementary Information (SI) for RSC Chemical Biology.
This journal is © The Royal Society of Chemistry 2026

Supporting Information

A nonavalent BODIPY with a multivalent arrangement of α-mannosides enables lectins recognition in fluorescence-based assays

Giacomo Biagiotti,[a,§] Edvin Purić,[b,§] Jacopo Tricomi,[a] Janez Mravljak,[b] Stefano Cicchi,[a] Marco Laurati,[a,f] Yvette van Kooyk,[c] Fabrizio Chiodo,[c,d] Iztok Urbančič,[e] Marko Anderluh,[b,*] Barbara Richichi[a,*]

[a]*Department of Chemistry 'Ugo Schiff', University of Firenze, Via della Lastruccia 3-13, 50019 Sesto Fiorentino, Italy*
[b]*Department of Pharmaceutical Chemistry, Faculty of Pharmacy, University of Ljubljana, Aškerčeva cesta 7, 1000 Ljubljana, Slovenia*
[c]*Department of Molecular Cell Biology and Immunology, Amsterdam UMC, Vrije Universiteit Amsterdam, Amsterdam 1081 HV, The Netherlands.*
[d]*Institute of Biomolecular Chemistry, National Research Council (CNR), via Campi Flegrei, 34, 80078 Pozzuoli, Naples, Italy.*
[e]*Laboratory of Biophysics, Condensed Matter Physics Department, Jožef Stefan Institute, Jamova Cesta 39, Ljubljana, Slovenia*
[f] *Consorzio per lo Sviluppo dei Sistemi a Grande Interfase, 50019 Sesto Fiorentino (FI), Firenze, Italy*

E-mail: barbara.richichi@unifi.it
Marko.Anderluh@ffa.uni-lj.si

[§] G.B. and E.P. equally contributed to this work

Table of content

**Figure S1**	pg S4
**Figure S2**	pg S5
**Figure S3**	pg S6
**Figure S4**	pg S7
**Figure S5**	pg S8
**Figure S6**	pg S9
**Figure S7**	pg S10
**Figure S8**	pg S11
**Figure S9**	pg S12
**Figure S10**	pg S13
**Figure S11**	pg S14
**Figure S12**	pg S14
**Figure S13**	pg S15
**Figure S14**	pg S16
**Table S1**	pg S16
**Table S2**	pg S16
**Figure S15**	pg S17
**Figure S16**	pg S17
**Figure S17**	pg S18
**Figure S 18**	pg S19
**Scheme S1**	pg S19
**Synthesis of 8**	pg S20
**Synthesis of 9**	pg S20
**Synthesis of 1**	pg S20
**Scheme 2**	pg S21
**Synthesis of 4**	pg S21

Synthesis of 5 pg S21
Synthesis of 2 pg S22
Figure S19 pg S23
Figure S20 pg S24
Figure S21 pg S25
Figure S22 pg S26
References pg S26

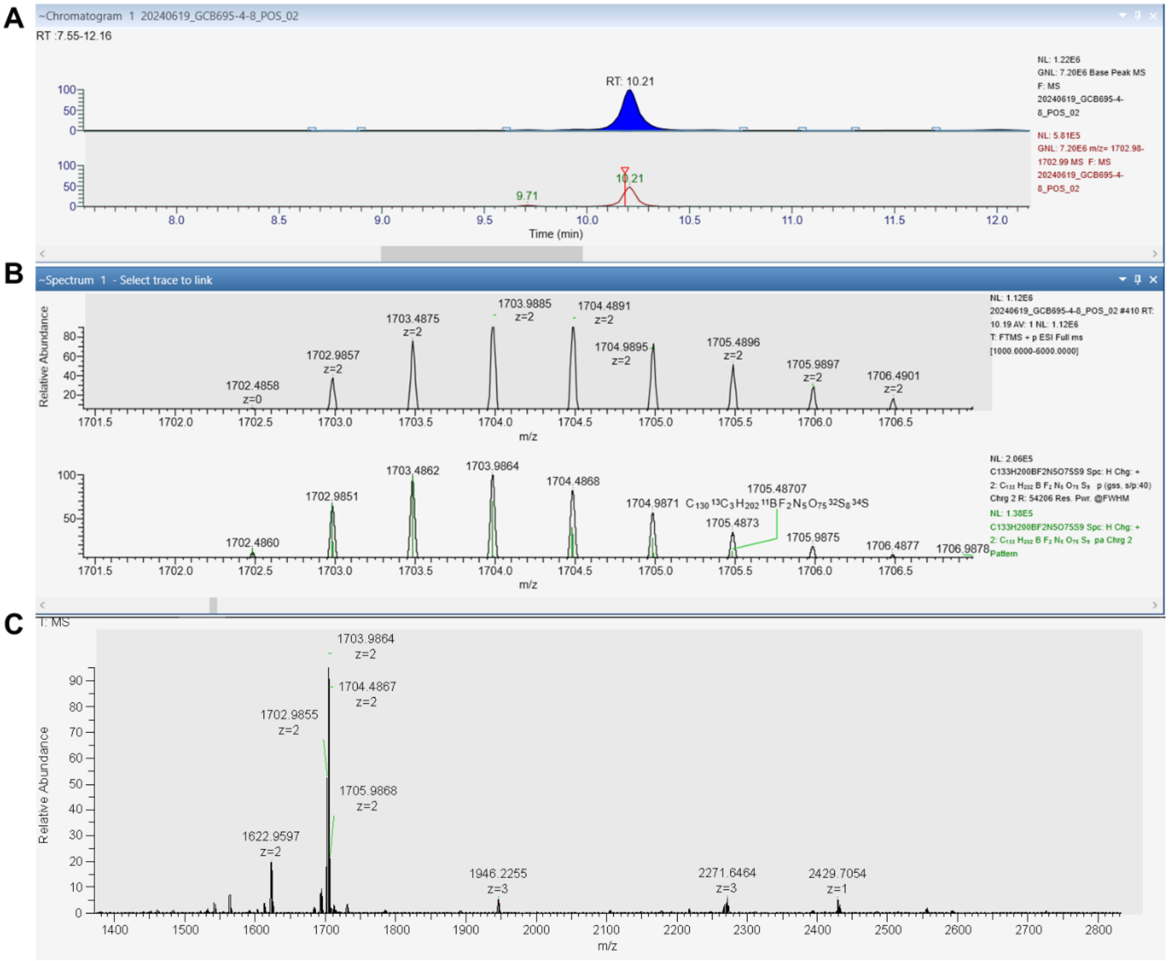

Figure S1. (**A**) LC-MS chromatogram of **Man$_9$-BODIPY**, retention time 10.2 minutes for **Man$_9$-BODIPY**; (**B**) isotopic pattern for the peak at retention time 10.2 minutes (upper panel calcd for $C_{133}H_{202}BF_2N_5O_{75}S_9$ [M+2H]$^{2+}$, lower panel measured); (**C**) HR-MS spectrum for the peak at retention time 10.2 minutes calcd. for $C_{133}H_{202}BF_2N_5O_{75}S_9$ [M+2H]$^{2+}$ 1703.9864 found 1703.9869, δ = -0.12 ppm.

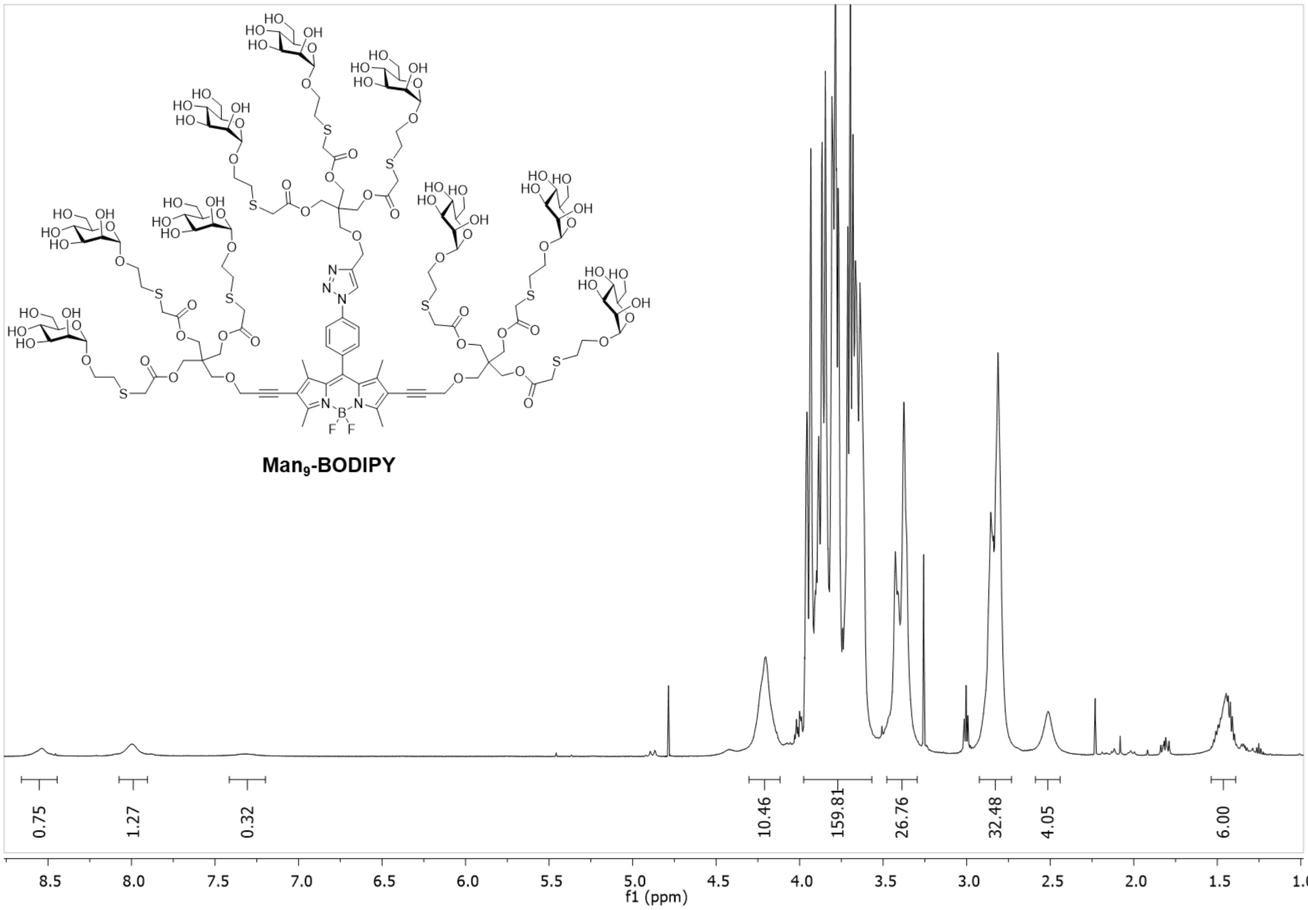

Figure S2. ^{1}H-NMR spectrum (D_2O, 400 MHz) of **Man$_9$-BODIPY**.

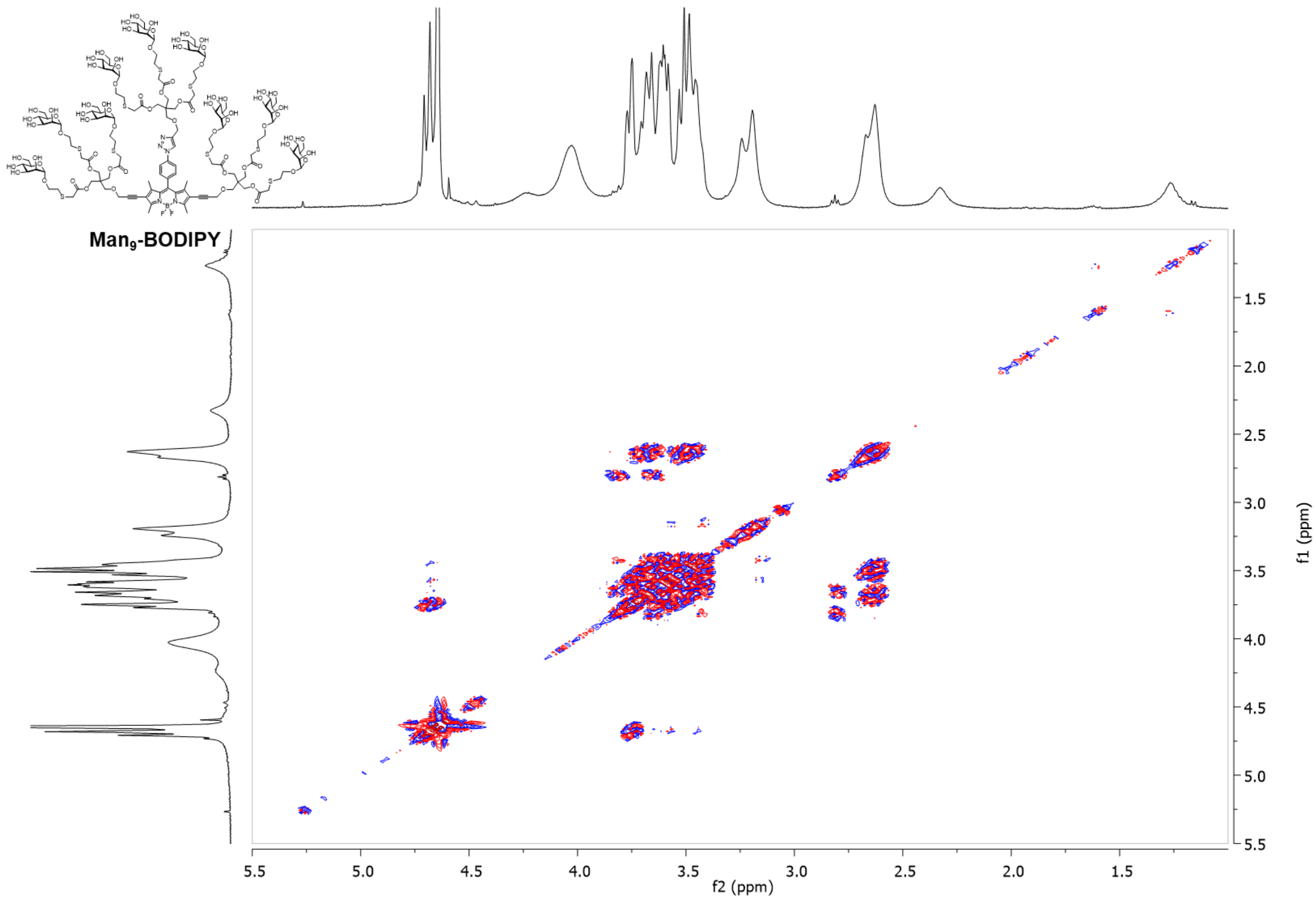

Figure S3. gCOSY-NMR spectrum (D_2O, 400 MHz) of **Man9-BODIPY**.

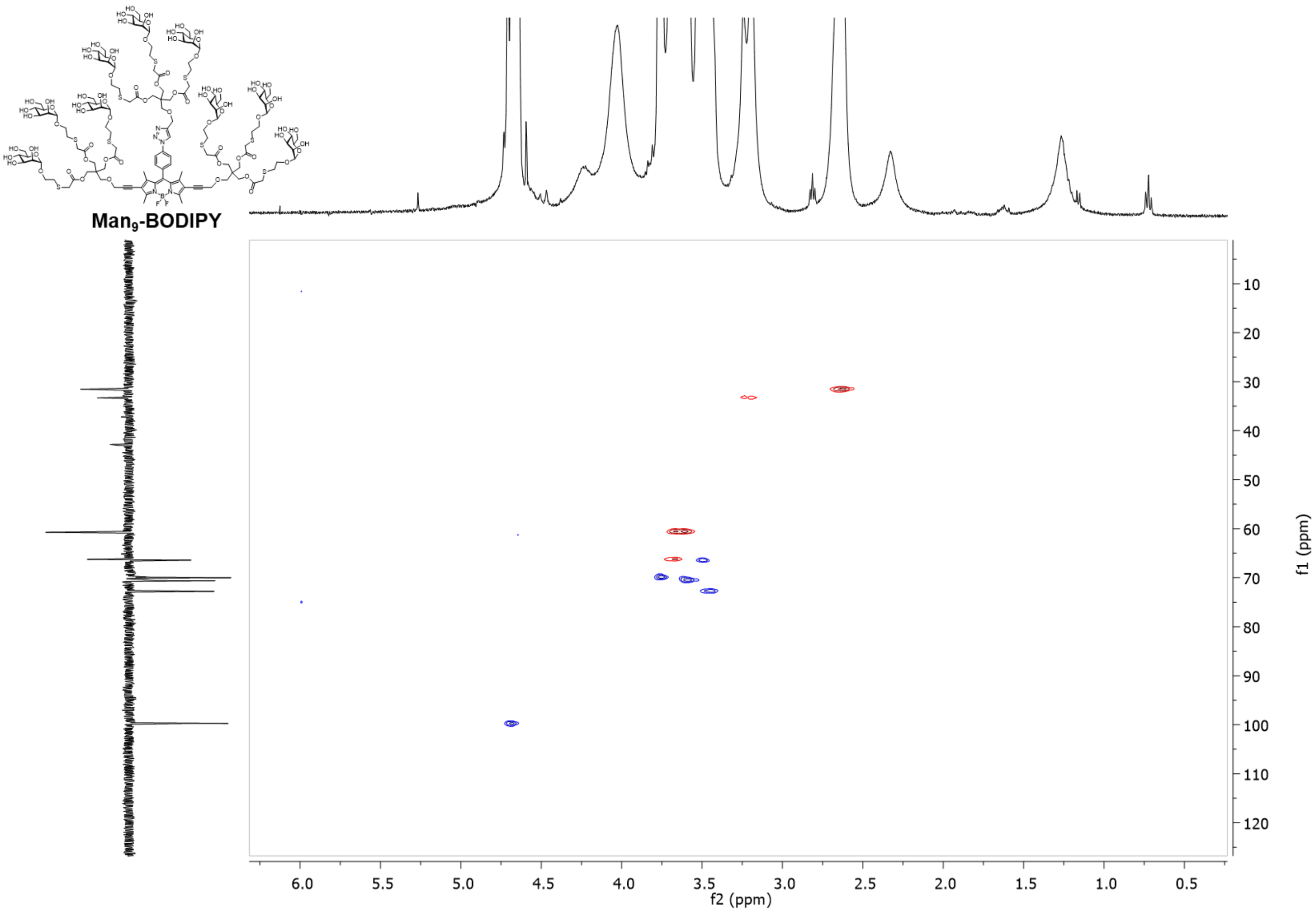

Figure S4. gHSQC-NMR spectrum (D_2O, 400 MHz) of **Man9-BODIPY**.

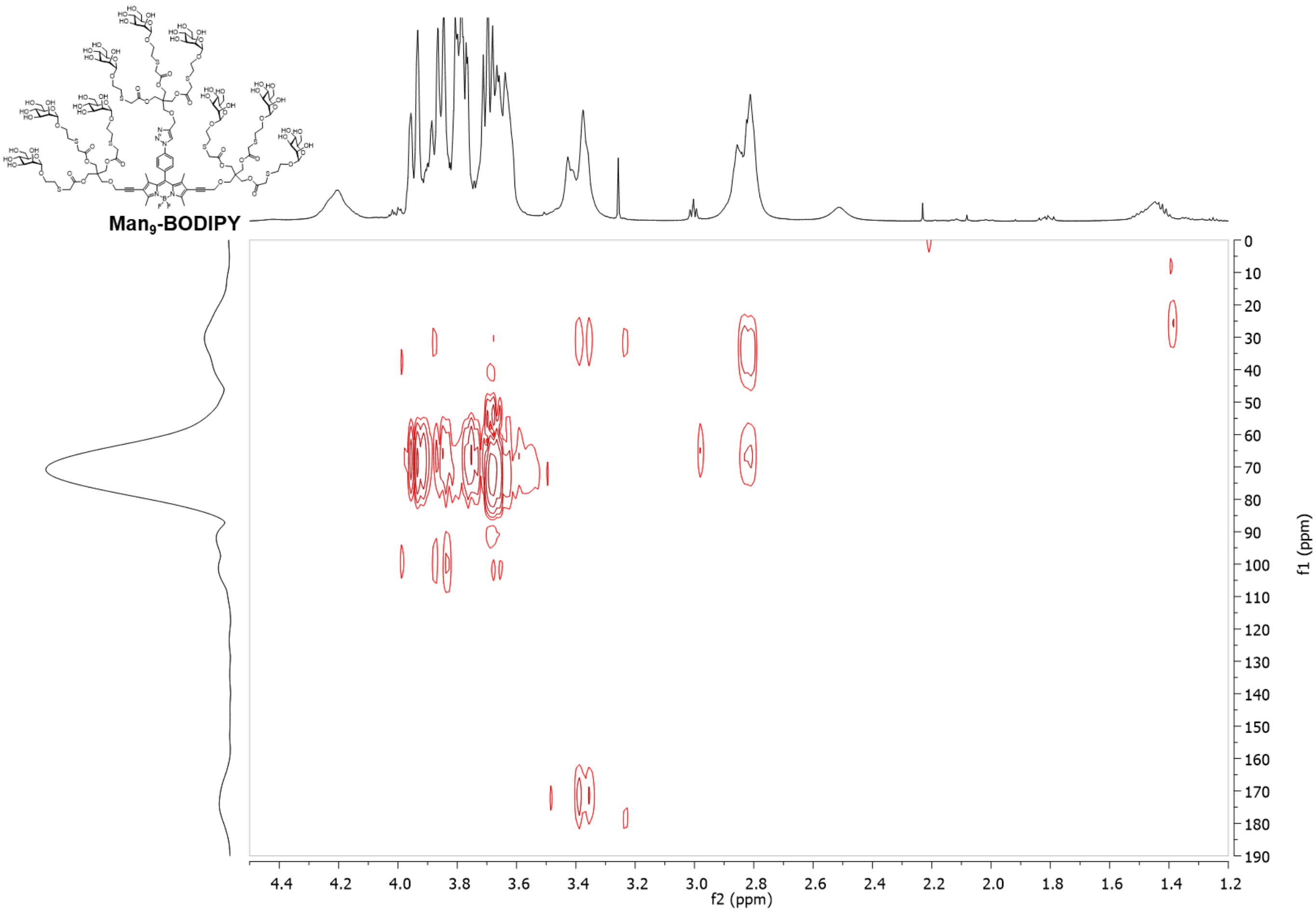

Figure S5. gHMBC-NMR spectrum (D_2O, 400 MHz) of **Man9-BODIPY**.

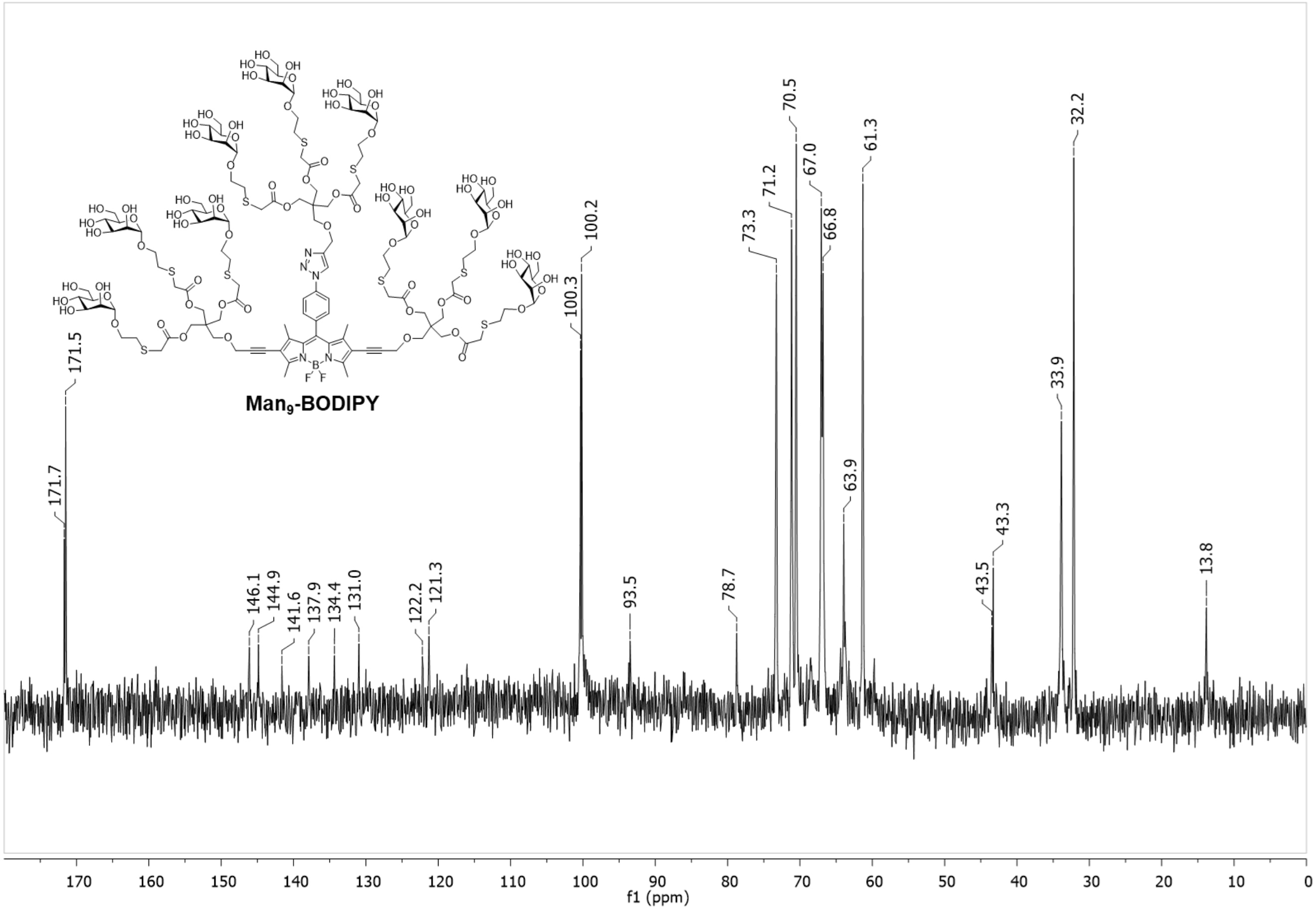

Figure S6. ^{13}C-NMR spectrum (D_2O, 100 MHz, 60°C) of **Man9-BODIPY**.

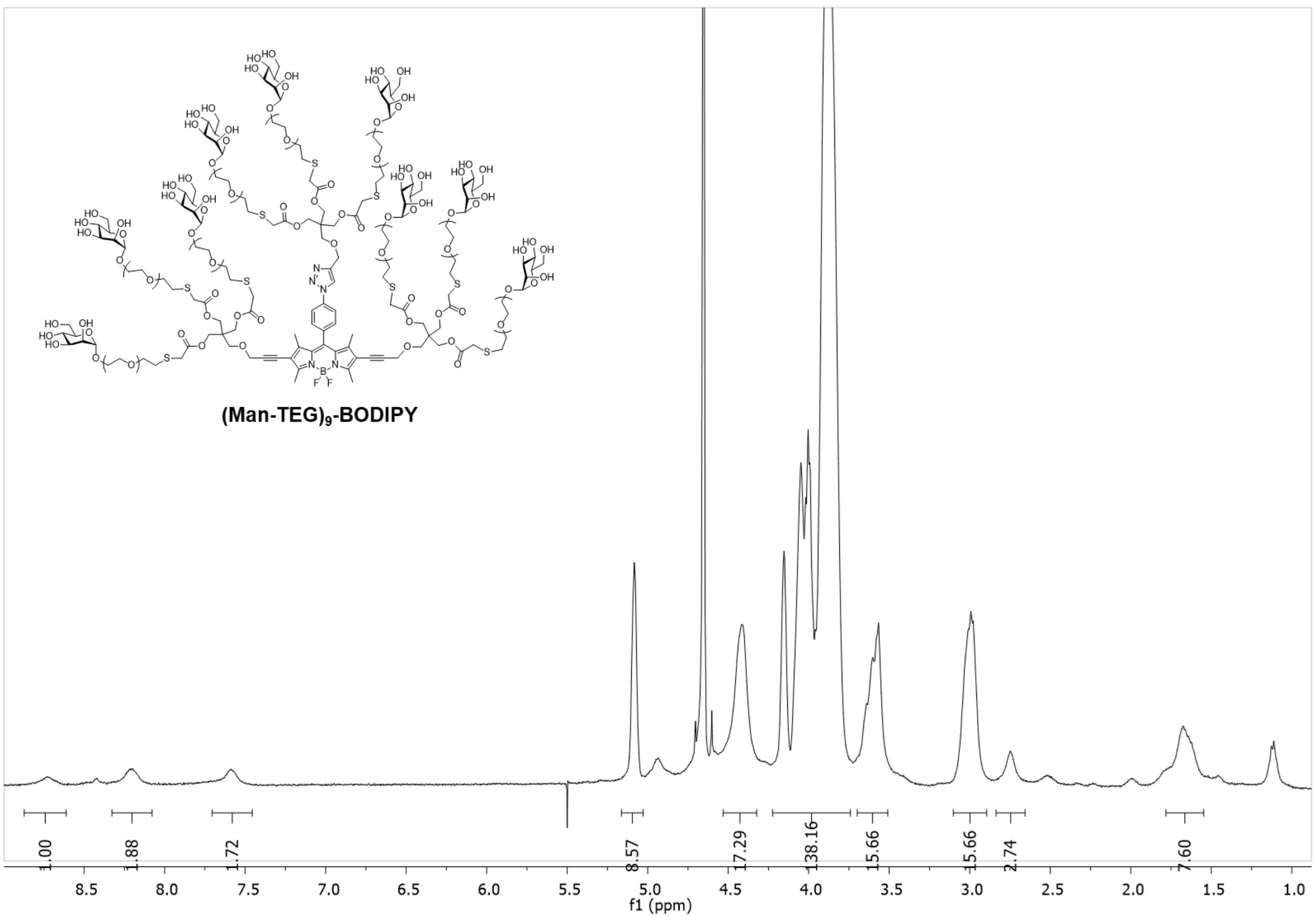

Figure S7. ^{1}H-NMR spectrum (D_2O, 400 MHz) of **(Man-TEG)$_9$-BODIPY**.

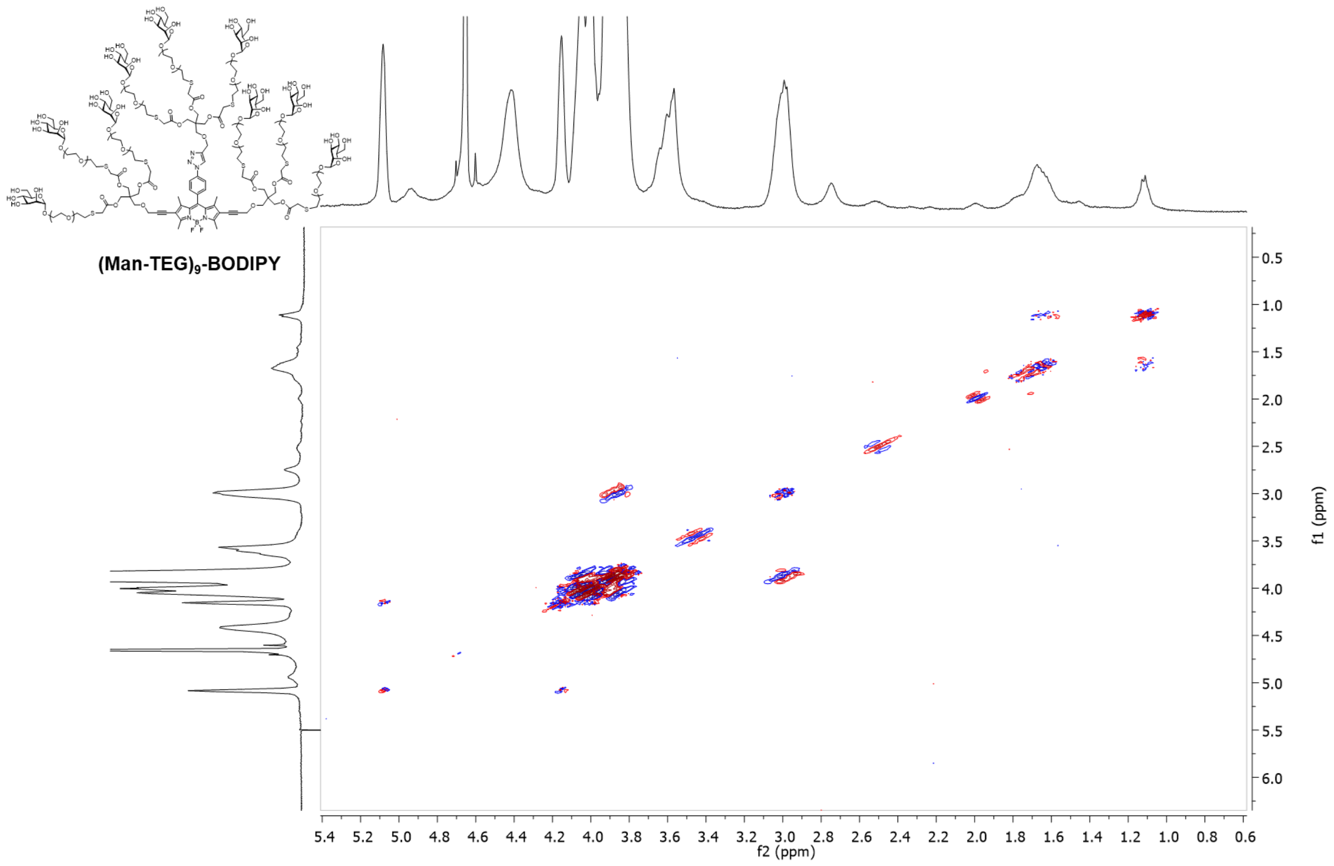

Figure S8. gCOSY-NMR spectrum (D_2O, 400 MHz) of **(Man-TEG)$_9$-BODIPY**.

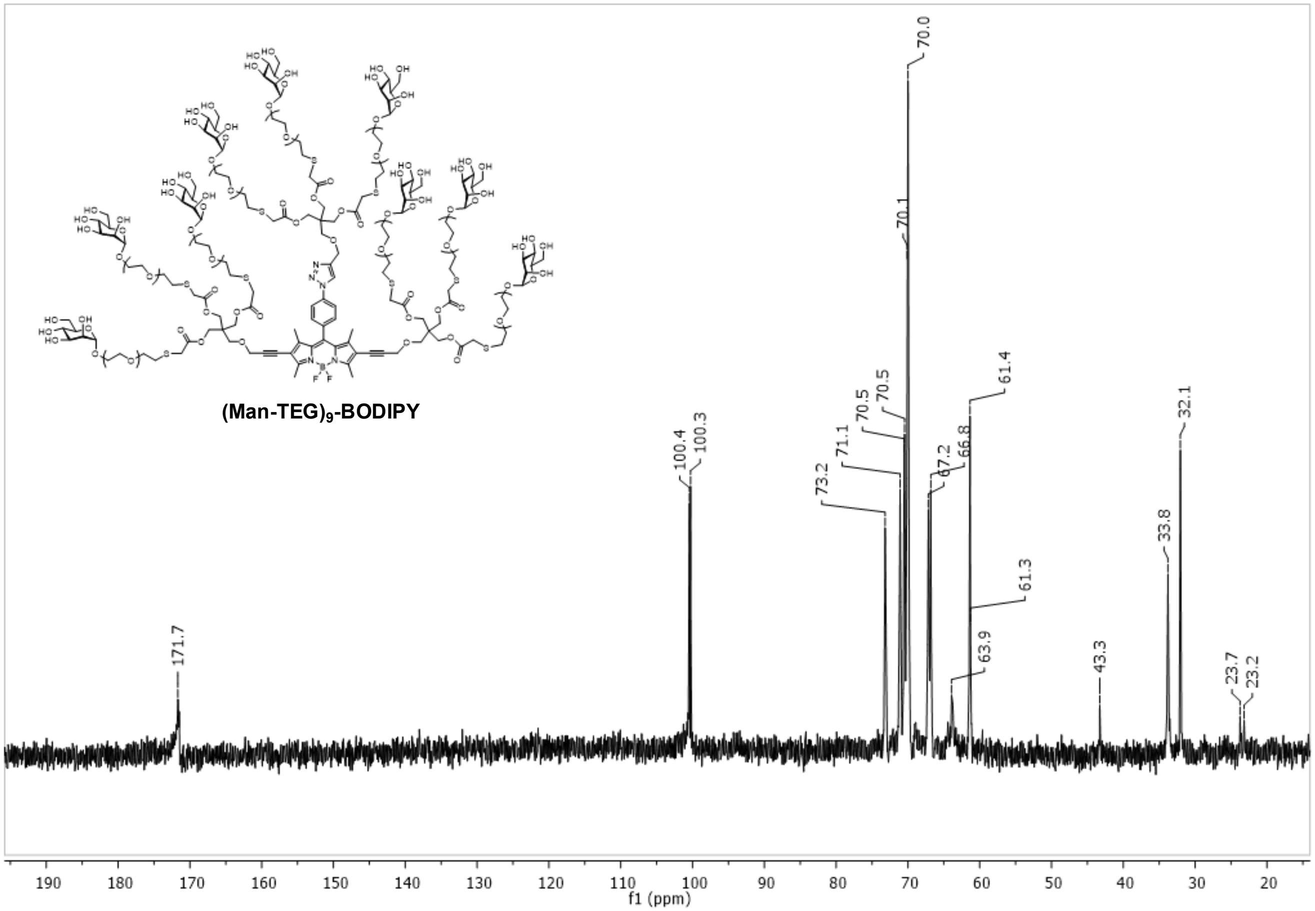

Figure S9. ^{13}C-NMR spectrum (D_2O, 100 MHz, 60°C) of **(Man-TEG)$_9$-BODIPY**.

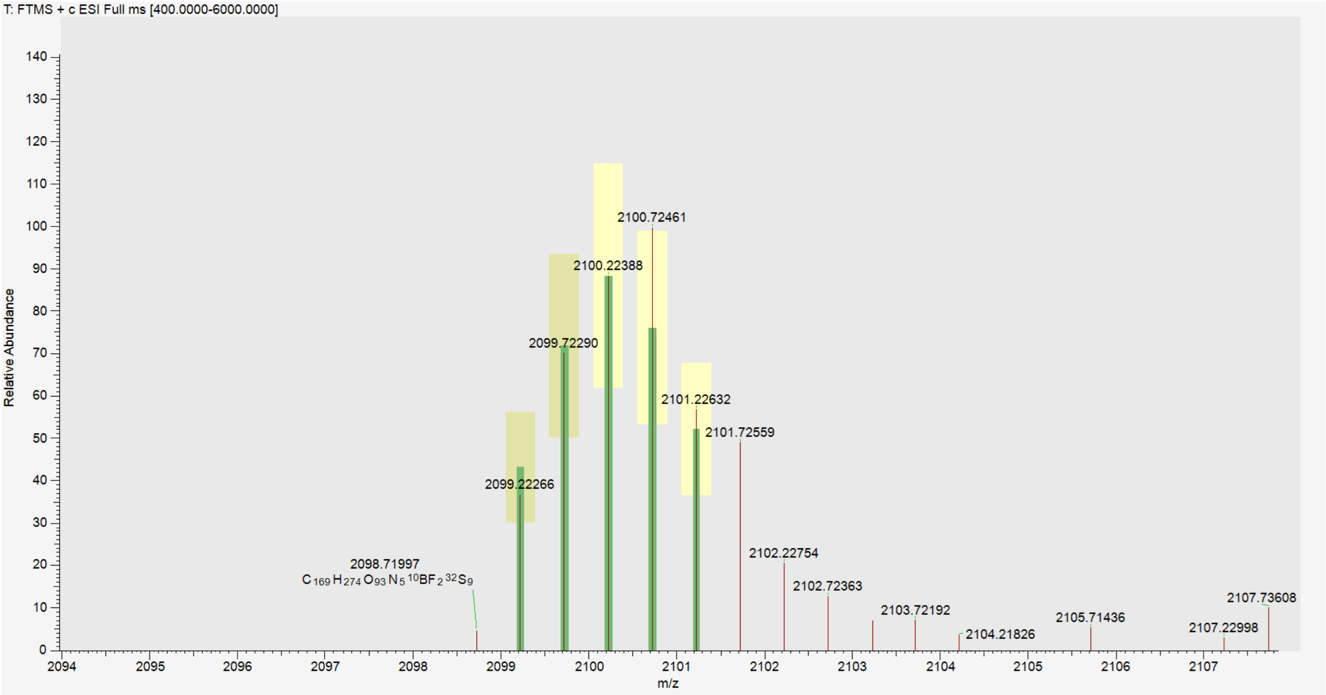

Peak Mass	Display Formula	S Fit	RDB	Delta [ppm]	Theo. mass	Rank	Combined Score	# Matched Iso.	# Missed Iso.	MS Cov. [%]	Pattern Cov. [%]
2098.71997	$C_{169}H_{274}O_{93}N_5{}^{10}BF_2{}^{32}S_9$	63.57524225	35	-0.91	2098.72188	4	86.48	5	0	87.76	100

Figure S10. Upper panel isotopic pattern for the **(Man-TEG)$_9$-BODIPY**, Lower panel HR-MS spectrum calcd. for $C_{169}H_{274}BF_2N_5O_{75}S_9$ $[M+2H]^{2+}$ 2098.72188 found 2098.71997, δ = -0.91 ppm.

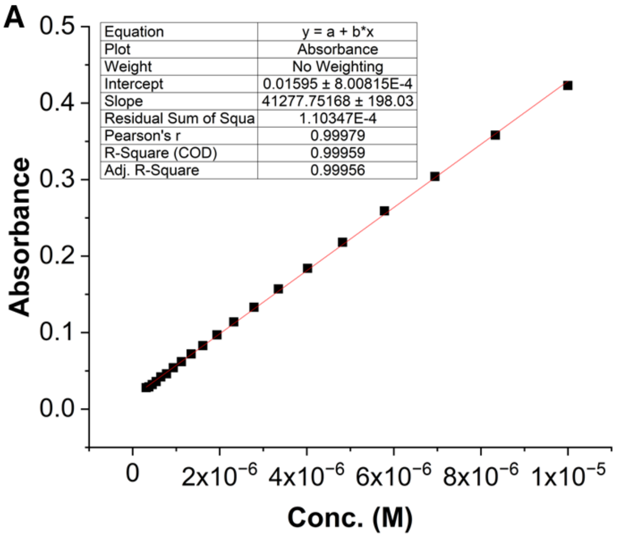

Figure S11. Molar extinction coefficients of **Man$_9$-BODIPY** in water 4.1 x 10^4 $M^{-1}cm^{-1}$.

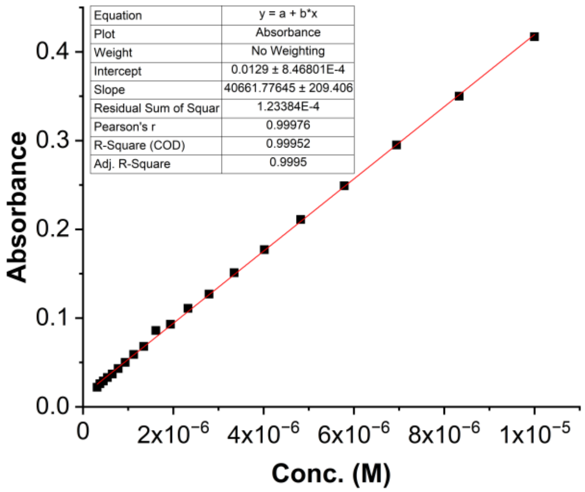

Figure S12. Molar extinction coefficients of **(Man-TEG)$_9$-BODIPY** in water 4.1 x 10^4 $M^{-1}cm^{-1}$.

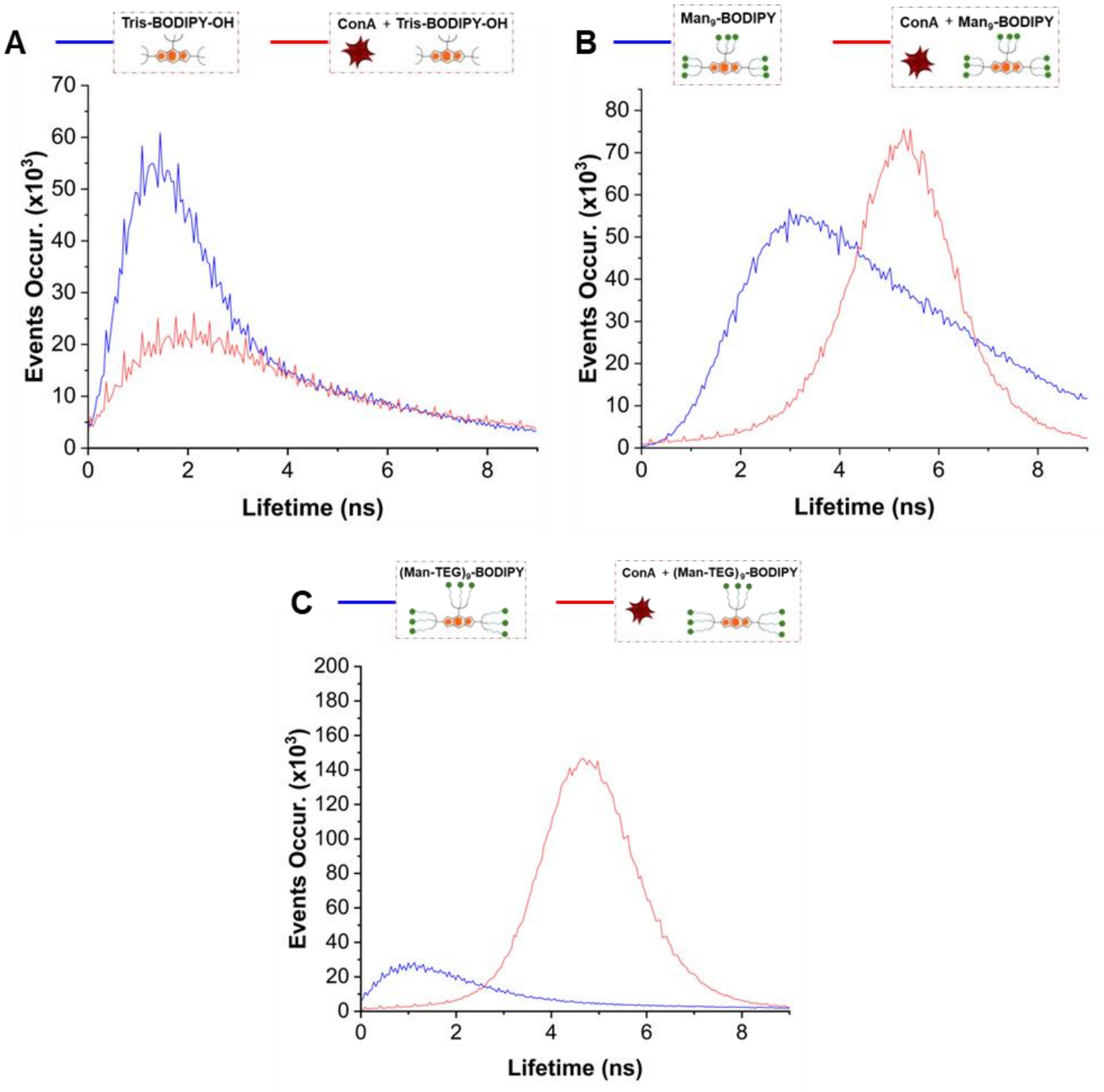

Figure S13. Lifetime histograms measured by FLIM. (**A**) **Tris-BODIPY-OH**, (**B**) **Man$_9$-BODIPY** and (**C**) **(Man-TEG)$_9$-BODIPY**. Blue curves: BODIPY solutions (500 nM in HEPES 25 mM pH 7.6, 1 mM $CaCl_2$); red curves: solutions of BODIPYs (500 nM) and ConA (500 nM) in HEPES 25 mM pH 7.6, 1 mM $CaCl_2$, 1 mM $MnCl_2$.

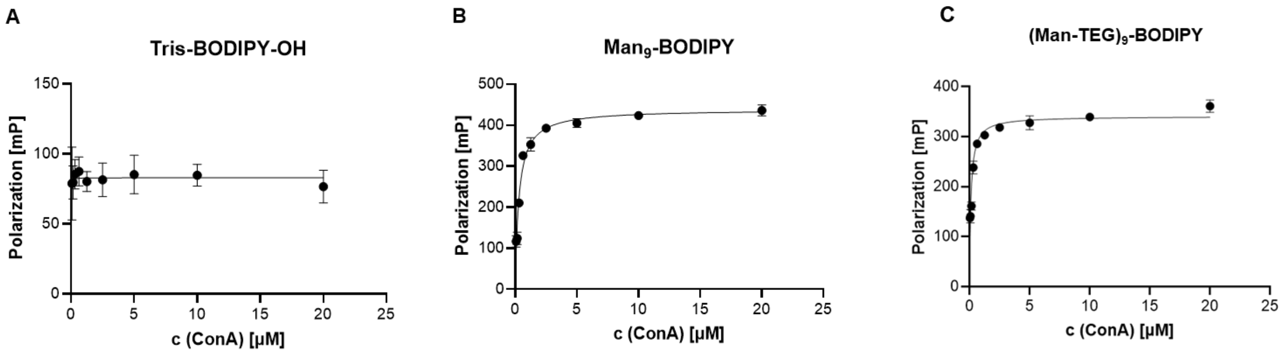

Figure S14. Binding curves of titration experiments using **Tris-BODIPY-OH** (**A**), **Man$_9$-BODIPY** (**B**), and **(Man-TEG)$_9$-BODIPY** (**C**) with increasing concentrations of ConA (0 – 25 μM). BODIPYs concentration: 0.5 μM.

Table S1. Binding properties of **Man$_9$-BODIPY** and **(Man-TEG)$_9$-BODIPY** *vs* ConA determined in a fluorescence polarisation assay (**Figure 5** and **Figure S16**).

**Man$_9$-BODIPY**	
	**Con A**
K_d [μM]	0.30 ± 0.04
**mP$_{max}$** [mP]	439 ± 27
**mP$_0$** [mP]	93.3 ± 12.9
**(Man-TEG)$_9$-BODIPY**	
K_d [μM]	0.12 ± 0.03
**mP$_{max}$** [mP]	340 ± 16
**mP$_0$** [mP]	94.0

Table S2. Binding properties of D-mannose against ConA determined in a competitive fluorescence polarisation assay.

K_d [mM]	1.35 ± 0.59
**mP$_{max}$**	432 ± 13
**mP$_0$**	113.9 ± 4.0

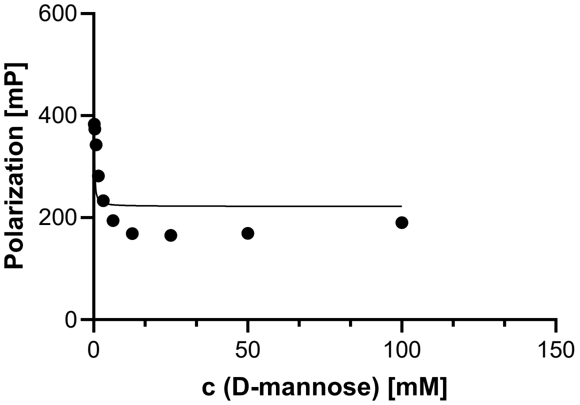

Figure S15. Binding curve of titration experiment. A competitive fluorescence polarization assay was done using a fixed concentration of **Man$_9$-BODIPY** (0.5 µM) and ConA (2 µM), which was titrated with increased concentration of D-mannose. The fluorescence polarization is expressed in millipolarization units (mP), while D-mannose concentration on x-axis is expressed in a linear fashion. Polarization curve shows dose-dependent reduction of the observed fluorescence anisotropy proving that the addition of D-mannose results in a competitive displacement of the probe.

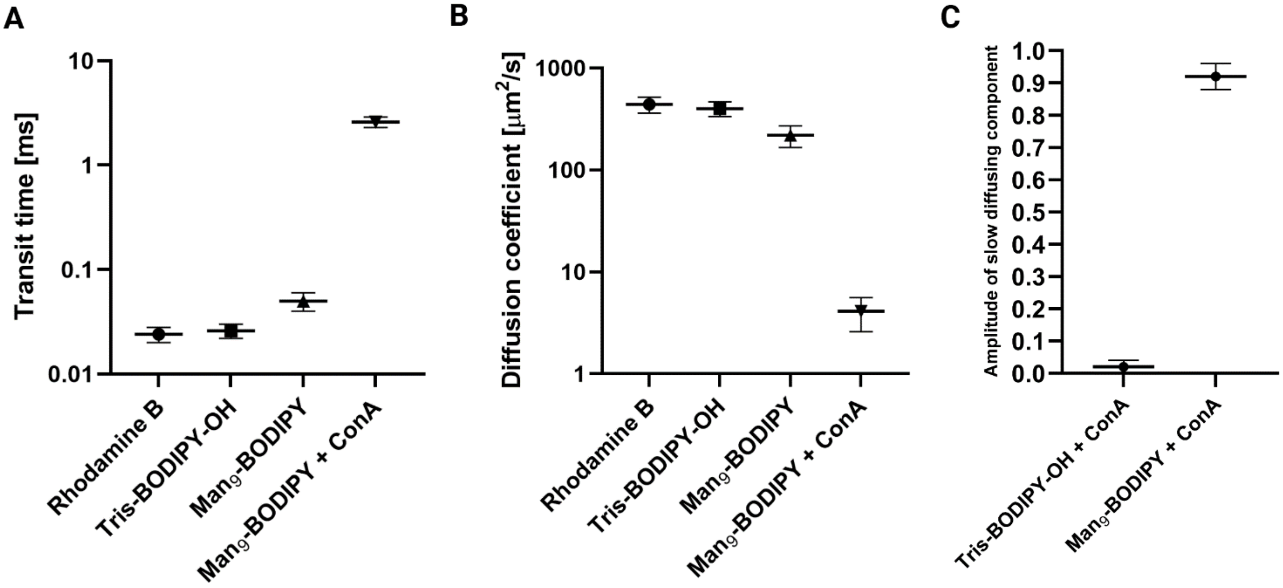

Figure S16. (**A**) Transit times of Rhodamine B, **Tris-BODIPY-OH** and **Man$_9$-BODIPY** with the addition of ConA, obtained by fitting the measured autocorrelation curves with the 3D diffusion

models. (**B**) Diffusion coefficients of Rhodamine B, which was used as a reference, **Tris-BODIPY-OH** and **Man$_9$-BODIPY** with the addition of ConA. According to the Stokes-Einstein equation, slower diffusion in the same environment indicates larger hydrodynamic radius of the diffusing entity; *i.e.* **Man$_9$-BODIPY** molecules diffuse more slowly in solution than **Tris-BODIPY-OH** due to additional Man groups and hence larger size. The diffusion coefficient is even lower when **Man$_9$-BODIPY** is bound to the ConA. (**C**) Amplitude of the slow diffusing component, describing the fraction of the probe bound to larger particles.

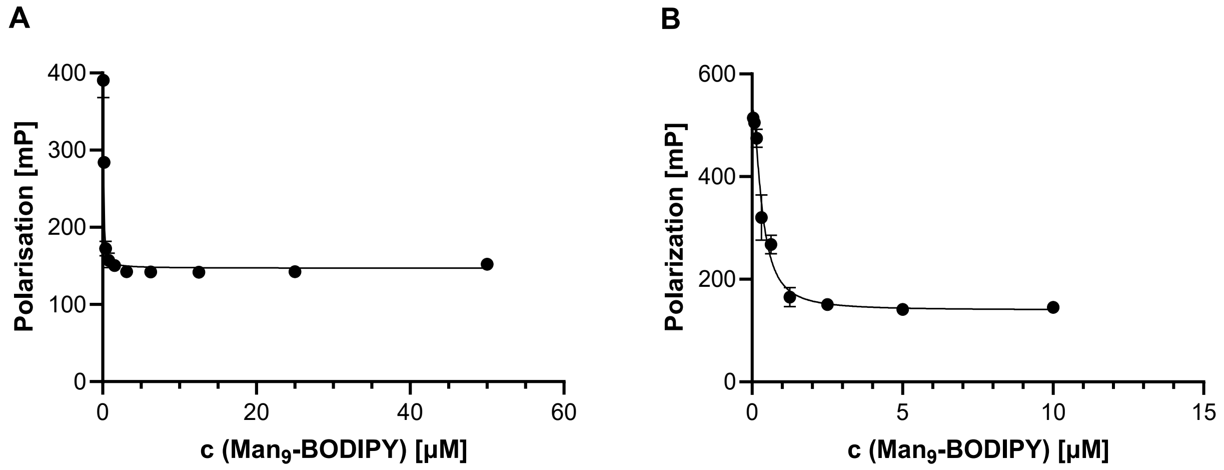

Figure S17. Binding curves from titration experiments showing the interaction of Langerin (**A**) and DC-SIGN (**B**) with increasing concentrations of **Man$_9$-BODIPY**.

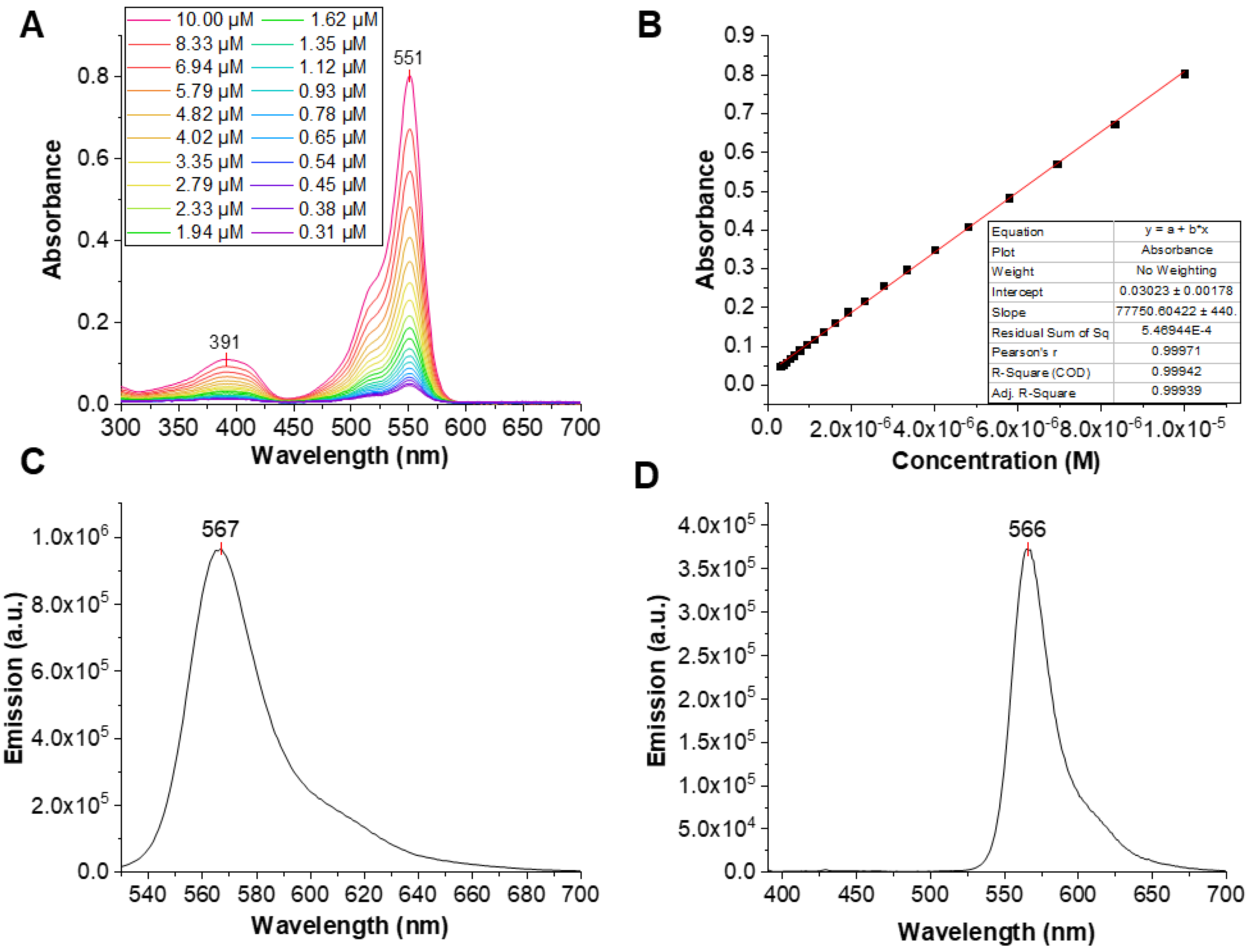

Figure S18. (**A**) Absorption spectra of solutions of **6** in dichloromethane at different concentrations; (**B**) Molar extinction coefficients of **6** in dichloromethane 7.8x10^4 M^{-1}cm^{-1}; (**C**) and (**D**) emission spectra of **6** (1 μM) in dichloromethane upon excitation at λ_{exc} = 520 nm (**C**) and 380 nm (**D**).

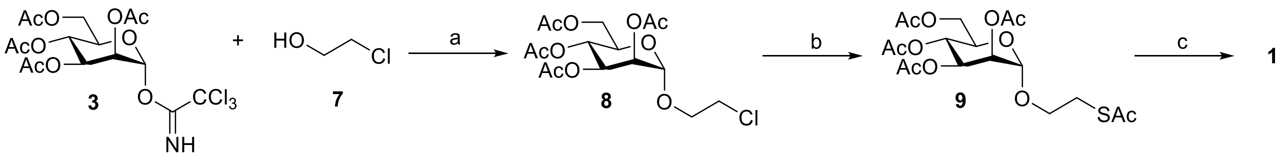

Scheme S1. Synthesis of α-mannoside **1**. a) (i) trifluoromethanesulfonate in dichlorometane 0-20 °C, 1 h; (ii) pyridine, acetic anhydride, *N,N*-4-dimethylaminopyridine, 1 h r.t. Yield: 75% over

two steps; b) potassium thioacetate, DMF, r.t., 24 h. Yield: 95%; c) (i) sodium methoxide, methanol, r.t., 72 h, (ii) tributyl phosphine, water:isopropanol 1:1, r.t. 12 h.

Synthesis of 8. α-Mannoside **8** was prepared according to a previously reported protocol with minor modifications.[1] In a round bottom flask, the glycosyl donor **3**[2] (1.83 g, 3.74 mmol) and the commercially available glycosyl acceptor **7** (602 mg, 7.48 mmol) were dried under vacuum for 30'. The mixture was dissolved in dry dichloromethane (6 mL) and cooled at 0°C, then trimethylsilyl trifluoromethanesulfonate (68 µL, 0.374 mmol) was added and the mixture was stirred for 5' at 0°C and then warmed at room temperature. After 1 h, dry triethylamine (155 µL, 1.12 mmol) was added and the crude was acetylated under standard conditions by adding to the reaction mixture, pyridine (606 µL, 7.48 mmol), acetic anhydride (707 µL, 7.48 mmol) and *N*-*N*-4-dimethylaminopyridine (91 mg, 0.74 mmol). After 1 h at room temperature, the reaction mixture was diluted with dichloromethane (200 mL), then the solution was washed with a saturated solution of ammonium chloride (2 x 15 mL), and with brine (1 x 15 mL). The organic phase was dried over sodium sulfate, filtered and the solvent removed under vacuum. The crude was purified by flash chromatography column (petroleum ether: ethyl acetate 3:1, *r.f.* = 0.3) to afford the α-mannoside **8** (1.14 g, 75 %) as a gummy solid. ^{1}H-NMR (200 MHz, $CDCl_3$): *δ* 5.51–5.19 (m, 3H, $H_{2\text{-}3\text{-}4}$), 4.87 (d, *J* = 1.6 Hz, 1H, H_1), 4.33-4.24 (A part of an ABX system, *J* = 12.5 Hz, *J* = 5.7 Hz, 1H, H_6), 4.21-4.04 (m, 2H, H_5, $H_{6'}$), 3.99-3.77 (m, 2H, CH_2-O), 3.68 (t, *J* = 5.6 Hz, 2 H, CH_2-Cl), 2.15 (s, 3H, OAc), 2.09 (s, 3H, OAc), 2.04 (s, 3H, OAc), 1.99 (s, 3H, OAc). Experimental data were in agreement with previously reported data.[1]

Synthesis of 9. Compound **9** was prepared according to a previously reported protocol.[1] Yield: 95%. ^{1}H NMR (400 MHz, $CDCl_3$) δ: 5.36 – 5.20 (m, 3H, $H_{2\text{-}3\text{-}4}$), 4.82 (d, *J* = 1.6 Hz, 1H, H_1), 4.29-4.24 (A part of an ABX system, *J* = 12.4 Hz, *J* = 5.6 Hz, 1H, H_6), 4.12-4.08 (B part of an ABX system, *J* = 12.0 Hz, *J* = 2.4 Hz, 1H, H_6), 4.06 – 3.99 (m, 1H, H_5), 3.80 – 3.74 (m, 1H, O-CH_2), 3.65 – 3.59 (m, 1H, O-CH_2), 3.11 (t, *J* = 6.4 Hz, 2H, S-CH_2), 2.36 (s, 1H), 2.15 (s, 3H, SAc), 2.09 (s, 3H, OAc), 2.05 (s, 3H, OAc), 1.99 (s, 3H, OAc). ^{13}C-NMR (50 MHz, $CDCl_3$) δ: 195.1, 170.6, 170.0, 169.8, 169.7, 97.5, 69.4, 68.9, 68.7, 66.9, 66.0, 62.4, 30.6, 28.5, 20.9, 20.7. Experimental data were in agreement with previously reported data.[1]

Synthesis of 1. Compound **9** was deacetylated following a previously reported protocols. [1,3] Briefly, compound **9** (414 mg, 1.09 mmol) was dissolved in methanol (17 mL) and sodium

methoxide (59 mg, 1.09 mmol) was added. The mixture was stirred at room temperature for 72 h, then the reaction was quenched by adding Dowex® marathon resin until pH 6.5. Then the resin was filtered off and the solvent removed under vacuum affording the crude disulfide (241 mg). The crude was then used, without any further purification, to obtain compound **1** following a previously reported protocol[3]. Briefly, the crude (241 mg) was dissolved in degassed isopropanol:water (1:1; 5 mL), then tributyl phosphine (101 mg, 0.5 mmol, 125 μL) was added and the mixture stirred for 12 h at room temperature. Then the solvent was removed under vacuum, and the crude was used for the next synthetic step without further purification.

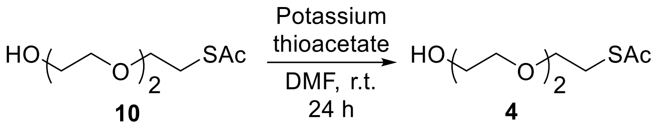

Scheme S2. Synthesis of **4**.

Synthesis of 4. In a round bottom flask, compound **10**[4] (1.4 g, 6.1 mmol) was dissolved in dimethylformamide (20 mL), then potassium thioacetate (4.2 g, 18 mmol) was added under nitrogen atmosphere and the mixture stirred at room temperature for 24 h. Then, the mixture was diluted with dichloromethane (200 mL) and washed with water (2 x 20 mL), a saturated solution of sodium bicarbonate (1 x 20 mL), and brine (1 x 20 mL). The organic phase was dried over sodium sulfate, filtered and the solvent removed under vacuum. The crude was purified by flash chromatography column (dichloromethane:ethyl acetate 1:1, *r.f.* = 0.6) to afford **4** (822 mg, 65 %) as brown oil. ESI-MS: calcd. for $C_6H_{12}NaO_3S^+$ $[M+Na]^+$ 231.07, found 231.00. ^1H NMR (200 MHz, $CDCl_3$) δ: 3.81 – 3.55 (m, 10H), 3.10 (t, *J* = 6.4 Hz, 2H), 2.34 (s, 3H). NMR data agreed with the literature.[4]

Synthesis of 5. In a round bottom flask, the glycosyl donor **3** (875 mg, 1.78 mmol) and the glycosyl acceptor **4** (555 mg, 2.66 mmol) were dried under vacuum for 30'. The mixture was dissolved in dry dichloromethane (6 mL) and cooled at 0°C, then trimethylsilyl trifluoromethanesulfonate (27.4 µL, 0.178 mmol) was added and the mixture was stirred for 5' at 0°C and then warmed at room temperature. After 1 h at room temperature, the reaction

mixture was diluted with dichloromethane (200 mL), then the solution was washed with a saturated solution of ammonium chloride (2 x 15 mL), and with brine (1 x 15mL). The organic phase was dried over sodium sulfate, filtered and the solvent removed under vacuum. The crude was purified by flash chromatography column (petroleum ether: ethyl acetate 1:1, *r.f.* = 0.3) to afford **5** (851 mg, 89 %) as a gummy solid. ESI-MS: calcd. for $C_{22}H_{34}NaO_{13}S^+$ $[M+Na]^+$ 561.16, found 561.20. ^{1}H-NMR (400 MHz, $CDCl_3$) δ: 5.35 (dd, J_{3-4} = 10.0 Hz, J_{3-2} = 3.2 Hz, 1H, H-3), 5.29 (ad, J = 10.0 Hz, 1H, H-4), 5.26 (dd, J_{2-3} = 3.2 Hz, J_{2-1} = 1.6 Hz, 1H, H-2), 4.86 (d, J_{1-2} = 1.6 Hz, 1H, H-1), 4.31-4.26 (A part of an ABX system, J = 12.4 Hz, J = 5.2 Hz, 1H, H-6a), 4.11 – 4.04 (m, 2H, H-6b, H-5), 3.84 – 3.78 (m, 1H, CH_2O), 3.71 – 3.56 (m, 10H, CH_2O), 3.09 (t, J = 6.5 Hz, 2H, CH_2S), 2.33 (s, 3H, SAc), 2.14 (s, 3H, OAc), 2.09 (s, 3H, OAc), 2.03 (s, 3H, OAc), 1.98 (s, 3H, OAc). ^{13}C-NMR (50 MHz, $CDCl_3$) δ: 195.5, 170.7, 170.0, 169.9, 169.7, 97.7, 70.6, 70.3, 70.0, 69.8, 69.5, 69.1, 68.4, 67.4, 66.1, 62.4, 30.6, 28.8, 20.9, 20.8, 20.7. NMR data agreed with data reported in the literature for this compound.[5,6]

Synthesis of 2. Compound **5** was deacetylated following a previously reported protocol. [1,3] Briefly, compound **5** (450 mg, 0.83 mmol) was dissolved in methanol (13.7 mL) and sodium methoxide (45 mg, 0.83 mmol) was added. The mixture was stirred at room temperature for 72 h, then the reaction was quenched by adding Dowex® marathon resin until pH 6.5. Then the resin was filtered off and the solvent removed under vacuum affording the crude (248 mg) disulfide. The crude was then used, without any further purification, to obtain compound **1** following a previously reported protocol.[3] Briefly, the crude (248.5 mg) was dissolved in degassed isopropanol:water (1:1; 3.1 mL), then tributyl phosphine (77 mg, 0.38 mmol, 95 μL) was added and the mixture stirred for 12 h. Then the solvent was removed under vacuum, and the crude was used for the next synthetic step without further purification.

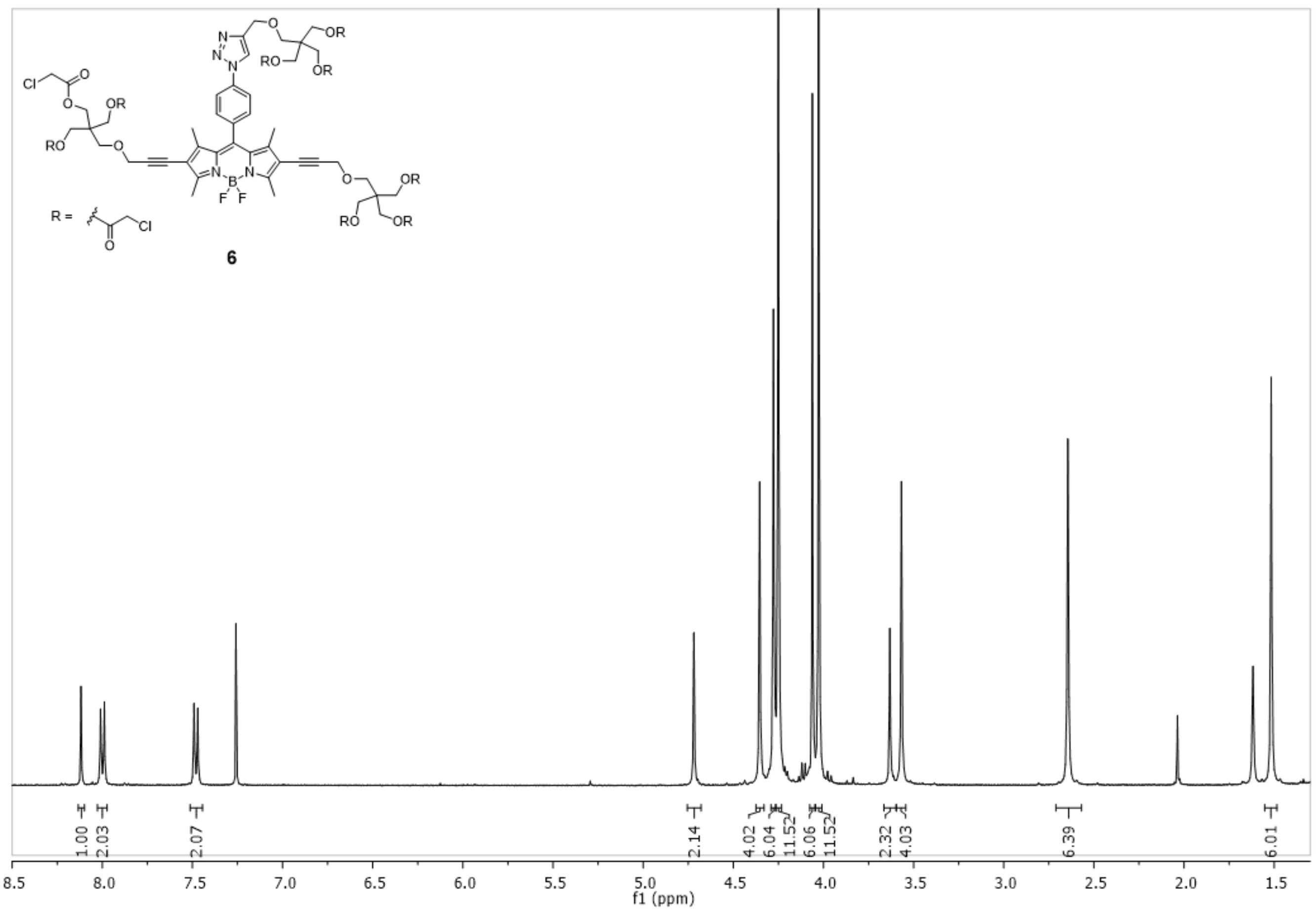

Figure S19. ^{1}H-NMR spectrum ($CDCl_3$, 400 MHz) of **6**.

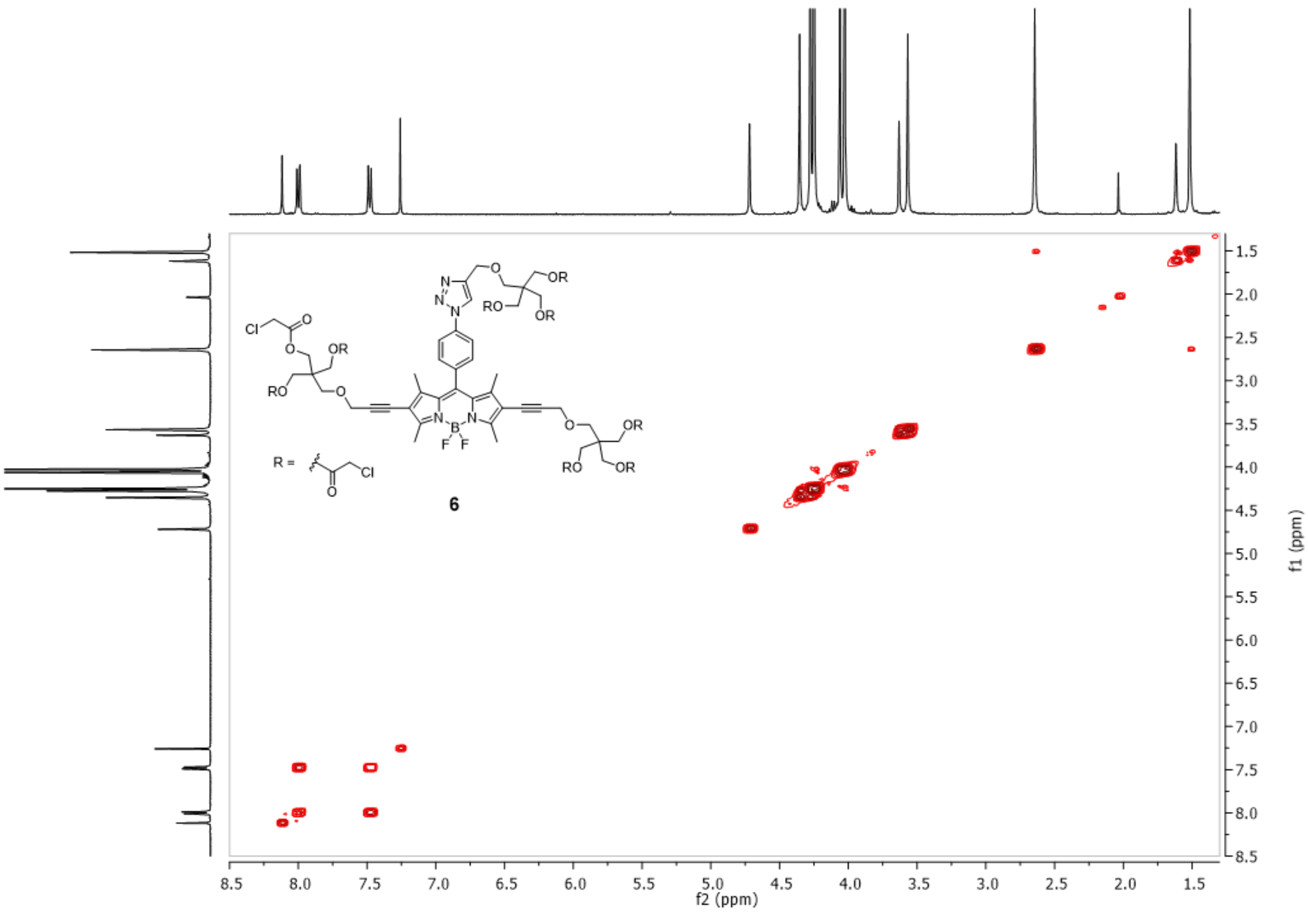

Figure S20. gCOSY-NMR spectrum ($CDCl_3$, 400 MHz) of **6**.

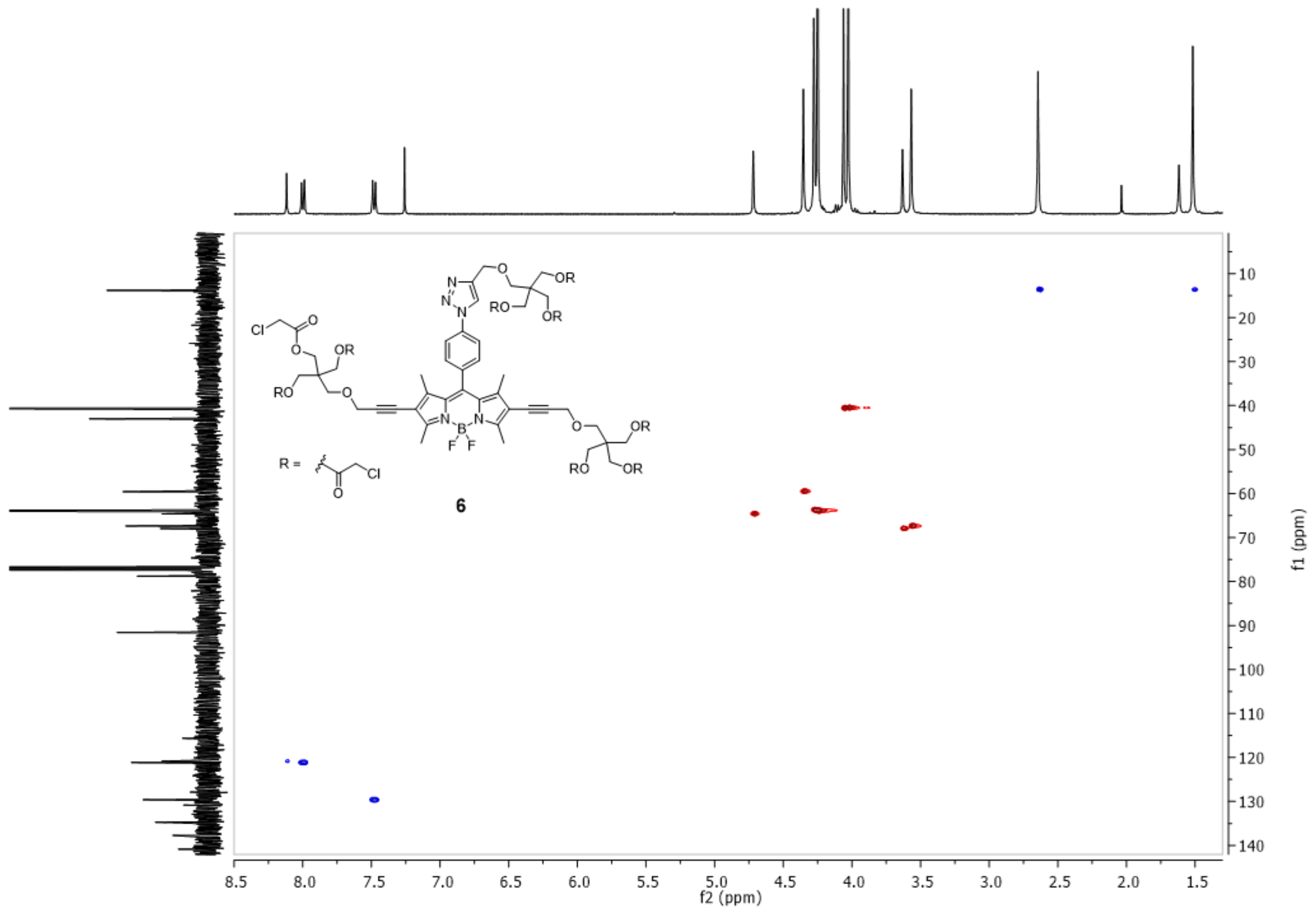

Figure S21. gHSQC-NMR spectrum ($CDCl_3$, 400 MHz) of **6**.

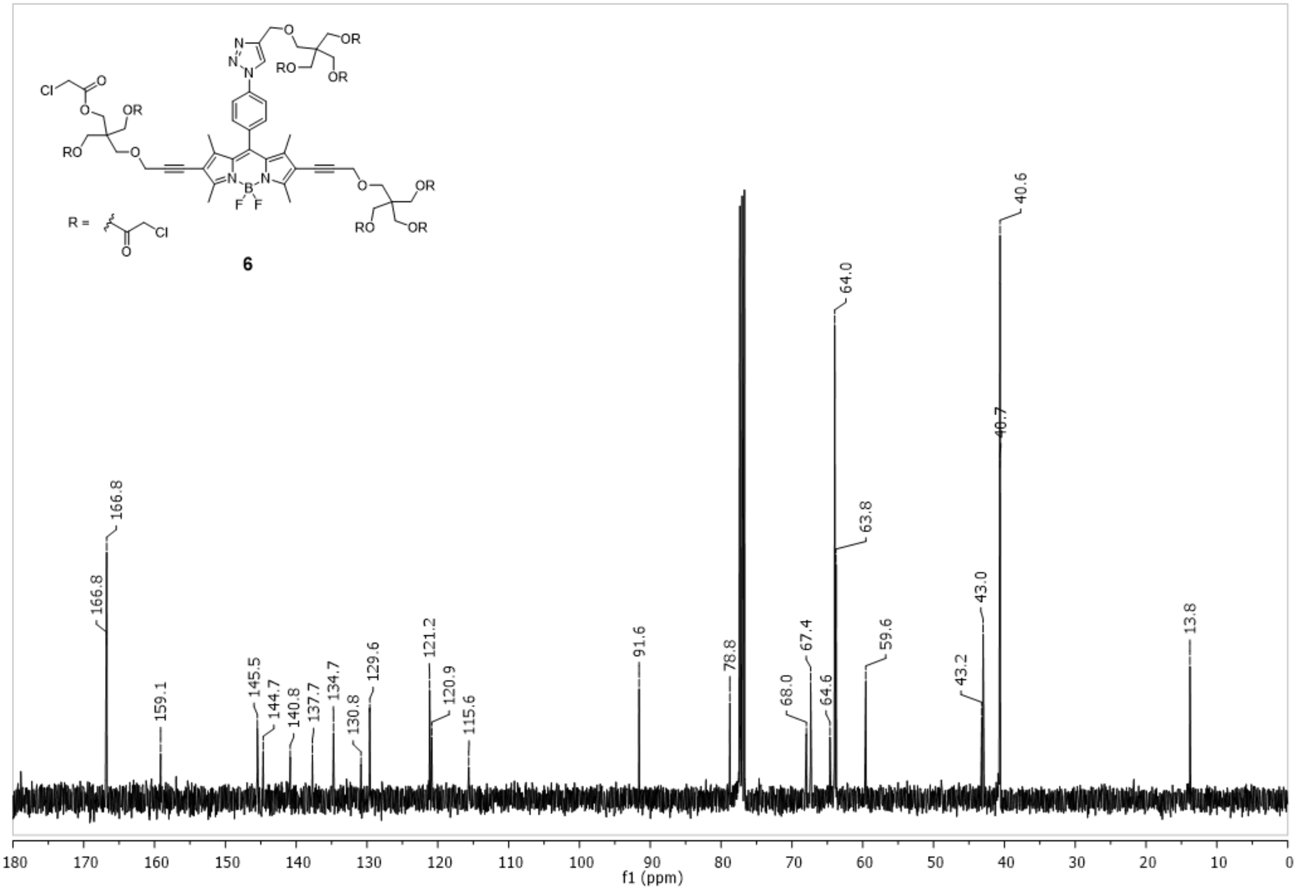

Figure S22. ^{13}C- NMR spectrum ($CDCl_3$, 100 MHz) of **6**.

References

1 O. Martínez-Ávila, K. Hijazi, M. Marradi, C. Clavel, C. Campion, C. Kelly and S. Penadés, *Chem. – A Eur. J.*, 2009, **15**, 9874–9888.

2 E. C. Stanca-Kaposta, D. P. Gamblin, E. J. Cocinero, J. Frey, R. T. Kroemer, A. J. Fairbanks, B. G. Davis and J. P. Simons, *J. Am. Chem. Soc.*, 2008, **130**, 10691–10696.

3 C. Grandjean, H. Gras-Masse and O. Melnyk, *Chem. - A Eur. J.*, 2001, **7**, 230–239.

4 J. Simonin, S. K. V. Vernekar, A. J. Thompson, J. D. Hothersall, C. N. Connolly, S. C. R. Lummis and M. Lochner, *Bioorg. Med. Chem. Lett.*, 2012, **22**, 1151–1155.

5 J. F. Longevial, K. El Cheikh, D. Aggad, A. Lebrun, A. van der Lee, F. Tielens, S. Clément, A. Morère, M. Garcia, M. Gary-Bobo and S. Richeter, *Chem. - A Eur. J.*, 2017, **23**, 14017–14026.

6 R. Kikkeri, B. Lepenies, A. Adibekian, P. Laurino and P. H. Seeberger, *J. Am. Chem. Soc.*, 2009, **131**, 2110–2112.
